# Structural and kinetic analyses of holothurian sulfated glycans suggest potential treatment for SARS-CoV-2 infection

**DOI:** 10.1016/j.jbc.2021.101207

**Published:** 2021-09-17

**Authors:** Rohini Dwivedi, Priyanka Samanta, Poonam Sharma, Fuming Zhang, Sushil K. Mishra, Pavel Kucheryavy, Seon Beom Kim, AyoOluwa O. Aderibigbe, Robert J. Linhardt, Ritesh Tandon, Robert J. Doerksen, Vitor H. Pomin

**Affiliations:** 1 Department of BioMolecular Sciences, University of Mississippi, Oxford, Mississippi, USA; 2 Department of Microbiology and Immunology, University of Mississippi Medical Center, Jackson, Mississippi, USA; 3 Center for Biotechnology and Interdisciplinary Studies, Rensselaer Polytechnic Institute, Troy, New York, USA; 4 Research Institute of Pharmaceutical Sciences, School of Pharmacy, University of Mississippi, Oxford, Mississippi, USA

**Keywords:** anticoagulation, fucosylated chondroitin sulfate, molecular modeling, nuclear magnetic resonance, surface plasmon resonance, viral inhibition, ACE2, angiotensin converting enzyme 2, aPTT, activated partial thromboplastin time, AT, antithrombin, BoSG, sulfated galactan from the red alga *Botryocladia occidentalis*, CS, chondroitin sulfate, DMEM, Dulbecco's modified Eagle medium, DS, dermatan sulfate, FDA, US Food and Drug Administration, FucCS, fucosylated chondroitin sulfate, GAG, glycosaminoglycan, GlcA, D-glucuronic acid, HS, heparan sulfate, HSQC, heteronuclear single quantum coherence, IbFucCS, FucCS isolated from *Isostichopus badionotus*, IbSF, *I. badionotus*-derived sulfated fucan, LvSF, sulfated fucan from the sea urchin *Lytechinus variegatus*, MD, molecular dynamics, PpFucCS, FucCS from the sea cucumber *Pentacta pygmaea*, RBD, receptor-binding domain, SA, streptavidin, SF, sulfated fucan, SG, sulfated galactan, SGP, spike glycoprotein, SPR, surface plasmon resonance, UFH, unfractionated heparin

## Abstract

Certain sulfated glycans, including those from marine sources, can show potential effects against SARS-CoV-2. Here, a new fucosylated chondroitin sulfate (FucCS) from the sea cucumber *Pentacta pygmaea* (PpFucCS) (MW ∼10–60 kDa) was isolated and structurally characterized by NMR. PpFucCS is composed of {→3)-β-GalNAcX-(1→4)-β-GlcA-[(3→1)Y]-(1→}, where X = 4S (80%), 6S (10%) or nonsulfated (10%), Y = α-Fuc2,4S (40%), α-Fuc2,4S-(1→4)-α-Fuc (30%), or α-Fuc4S (30%), and S = SO_3_^−^. The anti-SARS-CoV-2 activity of PpFucCS and those of the FucCS and sulfated fucan isolated from *Isostichopus badionotus* (IbFucCS and IbSF) were compared with that of heparin. IC_50_ values demonstrated the activity of the three holothurian sulfated glycans to be ∼12 times more efficient than heparin, with no cytotoxic effects. The dissociation constant (*K*_*D*_) values obtained by surface plasmon resonance of the wildtype SARS-CoV-2 spike (S)-protein receptor-binding domain (RBD) and N501Y mutant RBD in interactions with the heparin-immobilized sensor chip were 94 and 1.8 × 10^3^ nM, respectively. Competitive surface plasmon resonance inhibition analysis of PpFucCS, IbFucCS, and IbSF against heparin binding to wildtype S-protein showed IC_50_ values (in the nanomolar range) 6, 25, and 6 times more efficient than heparin, respectively. Data from computational simulations suggest an influence of the sulfation patterns of the Fuc units on hydrogen bonding with GlcA and that conformational change of some of the oligosaccharide structures occurs upon S-protein RBD binding. Compared with heparin, negligible anticoagulant action was observed for IbSF. Our results suggest that IbSF may represent a promising molecule for future investigations against SARS-CoV-2.

The pandemic caused by severe acute respiratory syndrome coronavirus (SARS-CoV-2) has made COVID-19, undoubtedly, a prime health concern worldwide nowadays (1, 2). Remdesivir is the only currently approved anti-COVID-19 drug (3). However, the US Food and Drug Administration (FDA), under its Coronavirus Treatment Acceleration Program, has approved emergency use authorization of certain other drugs for symptomatic treatment along with vaccines. With the massive COVID-19 vaccination drive rolling across the globe, treatment still heavily relies on administering drugs already approved by the FDA and using supportive care modalities (4, 5). Potential drug candidates with promising anti-SARS-CoV-2 activity are urgently needed as a supplemental mode of treatment that together with the current vaccine programs could control the disease spread effectively.

SARS-CoV-2 infection is primarily initiated by binding of SARS-CoV-2 spike glycoproteins (S-protein) to the heparan sulfate (HS) proteoglycans present on the host cell surfaces (6, 7). This molecular mechanism of virus–host interaction in the initial stages of the coronavirus infection has been described in many other viruses (8), for example, parainfluenza (9), adenovirus (10, 11, 12), human immunodeficiency virus (HIV) (13, 14), cytomegalovirus (12, 15), and herpes simplex virus (16), among others. In the case of SARS-CoV-2, interaction of the viral particle with HS and the human angiotensin converting enzyme 2 (ACE2) (17, 18, 19, 20, 21, 22) leads to virus fusion with the cellular membrane and eventual infection. The emergence of new SARS-CoV-2 variants with a key N501Y mutation in S-protein receptor-binding domain (RBD) (23) may lead to tighter binding to ACE2 receptor (24), thus causing higher infectivity (25) and increased human to human transmissibility (26). Interaction of SARS-CoV-2 with HS has been considered critical, and disruption of this intermolecular complex using exogenous heparin, its derivatives, or mimetics has been shown to potentially inhibit virus infectivity (6, 27, 28, 29).

Several studies have clearly shown the anti-SARS-CoV-2 effects of heparin and its derivatives in various model systems (27, 28, 29, 30). Heparin, however, being a potent anticoagulant is also associated with higher bleeding risk and heparin-induced thrombocytopenia (31, 32). Bleeding risk associated with heparin can lead to potential intracranial hemorrhage, which poses a practical downside to its possible acceptance as an anti-COVID-19 drug by the FDA (33). The presence of glycosaminoglycan (GAG)-binding-like motifs in the RBD and S2 proteolytic cleavage sites of SARS-CoV-2 S-protein (28, 34), along with the promising anti-SARS-CoV-2 activity of heparin executed through competitive inhibition of its binding to cell surface HS, suggests a strong rationale for exploring the anti-COVID-19 activity of other sulfated glycans (either GAGs or GAG mimetics).

Sulfated glycans are complex anionic polymers, composed of a linear chain of repeating sugar units (35, 36). They can be broadly classified into GAGs (37), such as HS, chondroitin sulfate (CS), dermatan sulfate (DS), heparin, and fucosylated chondroitin sulfate (FucCS), and GAG-like molecules (36, 38), including sulfated fucans (SFs) and sulfated galactans (SGs) (39). Apart from their structural and biological roles, sulfated glycans have very well-established therapeutic properties (35, 40) such as antiviral (12), antimicrobial (41), anticancer (42), and anticoagulant activities (43). The sulfation pattern of these polysaccharides makes them highly anionic and capable of specific interaction with different proteins involved in critical biological processes by electrostatic and stacking interactions (38, 39). The therapeutic relevance of these molecules has gained much attention during the COVID-19 pandemic owing to their significant antiviral potential against SARS-CoV-2 (27, 44, 45, 46, 47).

Here, we report the isolation and structural elucidation of a new fucosylated chondroitin sulfate (PpFucCS) from the body wall of a sea cucumber species *Pentacta pygmaea*. FucCSs, in general, are unique marine sulfated polysaccharides found exclusively in the body wall of sea cucumbers. These molecules are composed of alternating units of D-glucuronic acid (GlcA) and D-*N*-acetylgalactosamine (GalNAc) in the backbones with branching L-fucose (Fuc) units linked to the C3 position of the GlcA as the following {→3)-β-GalNAc-(1→4)-β-GlcA-[(3→1)X](1→}_n_ where X can be a branching monosaccharide (48) or disaccharide (49) of α-Fuc.

We also examine, for the first time, the anti-SARS-CoV-2 activity of the newly identified PpFucCS along with a known sulfated fucan (IbSF) (50) and fucosylated chondroitin sulfate (IbFucCS) (51) from *Isostichopus badionotus* and compare these with unfractionated heparin (UFH). Our results reveal promising anti-SARS-CoV-2 activity of all three holothurian sulfated glycans when examined in a pseudotyped SARS-CoV-2 baculoviral system. The anti-SARS-CoV-2 activity exhibited in the presence of these sulfated glycans seems to be mediated through competitive inhibition of the S-protein RBD binding with host surface HS, as demonstrated by competition results against UFH using surface plasmon resonance (SPR). All the glycans also exhibited strong binding to the S-protein N501Y mutant. Docking results indicate a similar binding mode, into a site close to N501, for the holothurian FucCS-containing oligosaccharide building blocks and heparin. Examination of the anticoagulant activity of all three holothurian sulfated glycans shows lower potency than UFH.

## Results

### Isolation and preliminary structural analyses of the polysaccharide-containing fractions from *P. pygmaea*

**Figure 1:**
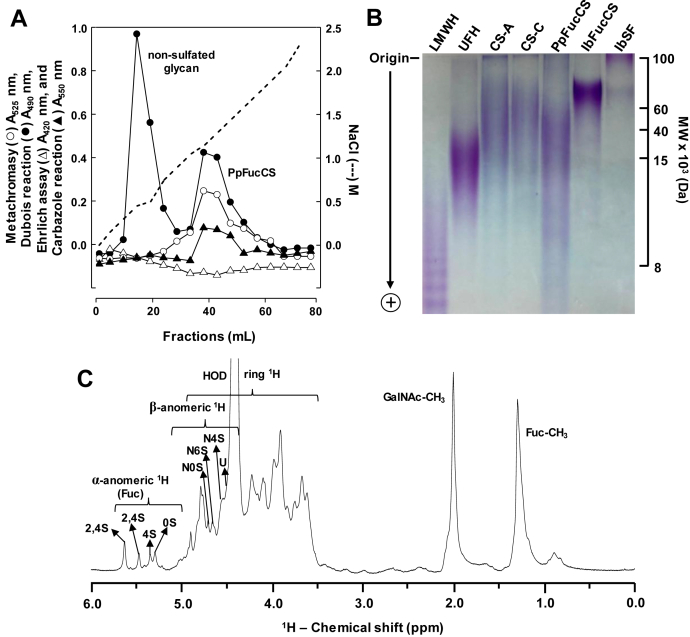
**Purification, electrophoretic mobility, and 1D**^**1**^**H NMR spectra of the polyanionic glycosidic fractions obtained from *Pentacta pygmaea*.***A*, crude polysaccharides (20 mg) obtained from the body wall of the sea cucumber *P. pygmaea* through nonspecific proteolytic (papain) digestion were fractionated in a DEAE cellulose column (2.5 × 20 cm) equilibrated with 100 mM sodium acetate buffer (pH 6.0). Multiple fractions (1 ml each) were obtained using a linear gradient of NaCl, from 0 to 3 M (---). Fractions were individually analyzed by Metachromasy using 1,9-dimethylmethylene blue (Abs 525 nm, ○) for the presence of sulfated glycan, by Dubois reaction (Abs 490 nm, •) for hexose, by Ehrlich assay (Abs 420 nm, Δ) for sialic acid, and by Carbazole reaction (Abs 550 nm, ▲) for uronic acid. The fractions corresponding to the respective peaks, labeled as nonsulfated glycan and the *P. pygmaea*-derived fucosylated chondroitin sulfate (PpFucCS), were pooled, dialyzed, and lyophilized for further characterization. *B*, molecular weight distribution of the purified PpFucCS was analyzed by polyacrylamide gel electrophoresis along with a series of molecular markers: low-molecular-weight heparin (LMWH) (∼8 kDa), unfractionated heparin (UFH) (∼15 kDa), chondroitin sulfate-A (CS-A) (∼40 kDa), chondroitin sulfate-C (∼60 kDa), and the sea cucumber *Isostichopus badionotus*-derived fucosylated chondroitin sulfate (IbFucCS) and sulfated fucan (IbSF). Samples (10 μg/each) were loaded on a 12% polyacrylamide gel and stained by 0.1% (w/v) toluidine blue (in 1% acetic acid) after electrophoretic migration. *C*, one-dimensional (1D) ^1^H NMR spectrum (δ_H_ expansion 6.0–0.0 ppm) of the purified PpFucCS were recorded in D_2_O, at 50 °C, on a 600 MHz Bruker NMR instrument. The NMR signals corresponding to characteristic peaks were properly labeled, as seen for the four composing α-fucose (Fuc) units (2,4S, 2,4S, 4S, and 0S) and the three composing β-N-acetylgalactosamine (GalNAc, N) units (4S, 6S, and 0S), all based on the diagnostic ^1^H signals present in the anomeric region, glucuronic acid (U) and GalNAc and Fuc methyl protons. S denotes sulfation in which the preceding number indicates the site or lack of occurrence.

The crude polysaccharide mixture obtained after nonspecific proteolytic (papain) digestion of the body wall of the sea cucumber *P. pygmaea* was subjected to anion-exchange chromatography on a DEAE cellulose column for purification, and fractions were monitored for the presence of sulfation, hexose, sialic acid, and uronic acid (Fig. 1*A*). Two peaks were obtained, one nonsulfated glycan and PpFucCS, starting elution, respectively, at 0.4 and 1.0 M NaCl concentrations (dashed line, Fig. 1*A*). The nonsulfated glycan was only positive for hexose (closed circles in Fig. 1*A*), whereas PpFucCS was positive for hexose, sulfation, and uronic acid content (closed circle, open circle, and closed triangle in Fig. 1*A*).

These glycans were further analyzed by polyacrylamide gel electrophoresis (PAGE) (Fig. 1*B*) and one-dimensional (1D) ^1^H nuclear magnetic resonance (NMR) spectroscopy (Figs. S1 and 1*C*). Electrophoretic migration of PpFucCS, as compared with other sulfated glycans of known molecular weight (MW), including the FucCS and SF from *I. badionotus* (IbFucCS and IbSF) of ∼70 to 80 and >100 kDa, respectively, has indicated a MW distribution of PpFucCS between ∼10 and ∼60 kDa (Fig. 1*B*). 1D ^1^H NMR spectra of the nonsulfated glycan (Fig. S1) and PpFucCS (Fig. 1*C*) showed contrasting signal profiles. The low and high amounts of ^1^H signals resonating, respectively, at the downfield anomeric region (δ_H_ between 5.6 and 4.2 ppm) and upfield methyl region (δ_H_ between 2.1 and 0.5 ppm), in the nonsulfated glycan, indicate a simple structure in terms of monosaccharide and anomeric composition, but complex, at the same time, in terms of methylation content (Fig. S1). On the other hand, the 1D ^1^H NMR spectrum of PpFucCS shows a typical pattern of FucCS polysaccharides, composed of two sets of anomeric signals: one related to the branching α-Fuc units (δ_H_ between 5.7 and 5.2 ppm) and the other related to the backbone β-monosaccharides, GalNAc and GlcA (δ_H_ between 4.8 and 4.4 ppm). The upfield methyl region of PpFucCS was also simpler, with the expected two distinct peaks of GalNAc (δ_H_ at 2.0 ppm) and Fuc (δ_H_ at 1.3 ppm) (Fig. 1*C*), as commonly seen in FucCS polysaccharides (52). NMR ^1^H analysis of PpFucCS must be performed at high temperature (50 °C) to avoid superimposition of the ^1^H signals from the β-units with the residual water (HOD) (Fig. S2).

### Structural elucidation of PpFucCS by two-dimensional NMR

**Figure 2:**
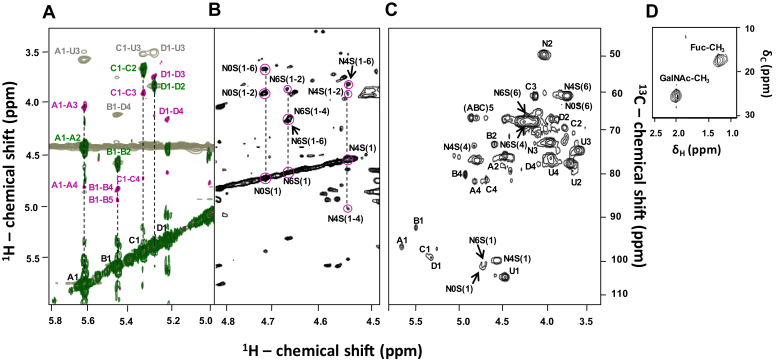
**2D**^**1**^**H-**^**1**^**H and**^**1**^**H-**^**13**^**C NMR spectra of the composing units of PpFucCS.***A*, the overlayed COSY (*green*), TOCSY (*magenta*), and 150 ms mixing time NOESY (*gray*) spectral strip from the anomeric region (δ_H_/δ_H_ expansions 5.8–5.0/6.0–3.25 ppm) shows the presence of four spin systems (*vertical dashed lines*) corresponding to four α-fucose (Fuc) units labeled as *A–D*, respectively, Fuc2,4S, Fuc2,4S, Fuc 4S, and Fuc. U denotes signals corresponding to glucuronic acid. *B*, δ_H_/δ_H_ expansions 4.8 to 4.5/6.0 to 3.25 ppm in TOCSY spectrum covering the anomeric region of N-acetylgalactosamine (GalNAc) units, showing the presence of three spin systems (*vertical dashed lines*) corresponding to the non-, 6-, and 4-sulfated GalNAc units labeled, respectively, as N0S, N6S, and N4S. Pairs of numbers between parentheses indicate position of the ^1^H nuclei assigned in the cross-peaks, marked by circles. *C* and *D*, ^1^H-^13^C HSQC spectrum (δ_H_/δ_C_ expansions 6.0–3.25/112.0–45.0 ppm, and δ_H_/δ_C_ expansions 2.5–1.0/30.0–10.0 ppm), showing all ^1^H-^13^C cross-peaks of PpFucCS. All 2D NMR spectra were acquired at 50 °C on a 600 MHz Bruker NMR instrument. Labels on ^1^H-^13^C cross-peaks of (*C*) and (*D*) follow the same patterns of (*A*) and (*B*).

The full δ_H_ and δ_C_ assignment of PpFucCS (Table 1) was achieved through a combination of multiple 2D NMR methods, all acquired at 50 °C (Fig. 2). Assignments were initiated using three-bond (short-range) ^1^H–^1^H connections of vicinal carbons in the hexose rings through correlation spectroscopy (COSY) (green spectrum, Fig. 2*A*, for α-Fuc units). Then, spin-system assignments (long-range) of ^1^H–^1^H connections were obtained by TOCSY (magenta spectrum, Fig 2*A*, for α-Fuc units, and Fig. 2*B*, for GalNAc units). The full notation of δ_H_ aided further assignments of both the through-space ^1^H–^1^H connectivities seen through NOESY (gray spectrum, Fig. 2*A*) and full δ_C_ in the ^1^H–^13^C heteronuclear single quantum coherence (HSQC) spectrum (Fig. 2, *C* and *D*). NOE build-up curves were generated for the α-Fuc-related NOE cross-peaks observed in PpFucCS to reduce the impact of spin-diffusion in the NOESY analysis (Fig. S3). The NOESY spectrum of 150 ms showed the best signal-to-noise ratio in the linear region of the NOE build-up curves (Fig. S3) and was, hence, selected for assignment (gray spectrum, Fig. 2*A*).

**Table 1:** ^1^H and^13^C chemical shifts (ppm) of composing units from *P. pygmaea*–derived fucosylated chondroitin sulfate (PpFucCS) and references

Unit	Source	^1^H1/^13^C1	^1^H2/^13^C2	^1^H3/^13^C3	^1^H4/^13^C4	^1^H5/^13^C5	^1^H6,^1^H6′/^13^C6	GalNAc-CH_3_
Fuc2,4S (A)	PpFucCS^a^	5.63/96.8	**4.45/75.5**	4.08/61.4	**4.78/81.6**	ND/66.5	1.3/16.2	-
Fuc2,4S (B)	PpFucCS^a^	5.47/92.4	**4.57/72.7**	3.87/67.0	**4.90/79.8**	4.80/66.4	1.3/16.2	-
Fuc2,4S	Reference^b^	5.69/96.9	**4.50/75.6**	4.13/67.2	**4.84/81.6**	4.90/66.7	1.36/16.4	-
Fuc4S (C)	PpFucCS^a^	5.34/98.9	3.75/68.5	3.98/68.9	**4.70/81.2**	ND	1.3/16.2	-
Fuc4S	Reference^b^	5.40/98.9	3.83/72.3	4.01/67.9	**4.75/81.6**	4.84/66.7	1.36/16.4	-
Fuc0S (D)	PpFucCS^a^	5.28/99.4	3.90/66.8	3.83/68.0	*4.15/78.0*	ND	1.3/16.2	-
Fuc	Reference^b^	5.28/97.9	4.30/72.5	3.99/72.3	4.16/72.5	4.15/69.2	1.36/16.4	-
GalNAc (N0S)	PpFucCS^a^	4.71/101.0	3.99/51.8	*3.92/72.3*	4.20/69.7	ND	4.05,3.75/61.4	2.01/22.9
GalNAc	Reference^c^	4.55/102.4	4.01/54.1	*3.91/74.6*	4.20/69.1	3.92/74.8	4.09,3.74/61.4	-
GalNAc4S (N4S)	PpFucCS^a^	4.53/100.0	3.99/51.8	*3.92/72.3*	**4.78/76.2**	ND	4.05,3.75/61.4	2.01/22.9
GalNAc4S	Reference^c^	4.55/102.4	4.01/54.1	*3.91/74.6*	**4.75/78.8**	3.92/74.8	4.09,3.74/61.4	-
GalNAc6S (N6S)	PpFucCS^a^	4.65/100.9	3.99/51.8	*3.92/72.3*	4.20/69.7	ND	**4.19,4.10/67.3**	2.01/22.9
GalNAc6S	Reference^c^	4.55/102.4	4.01/54.1	*3.91/74.6*	4.20/69.1	3.92/74.8	**4.26–4.19/67.6**	-
GlcA (U)	PpFucCS^a^	4.43/104.0	3.60/74.1	*3.68/77.4*	*3.90/76.4*	ND	-	-
GlcA	Reference^b^	4.48/104.0	3.67/74.3	*3.72/77.6*	*3.98/75.8*	4.07/72.5	-	-

Values in bold indicate sulfation sites. Values in italic indicate glycosylation sites.

Abbreviation: ND, not determined.

a The spectra were recorded at 600 MHz in 99.9% D_2_O at 50 °C. Chemical shifts are relative to external trimethylsilylpropionic acid 0 ppm for ^1^H and to methanol for ^13^C.

b Soares *et al*. (49).

c Niu *et al*. (101).

Comparison between the δ_H_ and δ_C_ values obtained through this series of 2D NMR spectra (Fig. 2) with reference values (Table 1) allowed us to elucidate the sulfation patterns and glycosidic bonds of all composing units in PpFucCS. The α-Fuc units ascribed as A, B, and C of anomeric ^1^H's with δ_H_ at 5.63, 5.47, and 5.34 ppm, respectively, show low-field ^1^H shifts (∼0.7 ppm) on both C2 and C4 sites (units A and B) or only on the C4 site (unit C), indicative of sulfation. In contrast, the α-Fuc unit ascribed as D, of anomeric ^1^H's with δ_H_ at the most upfield region, 5.28 ppm, shows high-field ^1^H shifts on both C2 and C4 sites, clearly indicating no sulfation. The relative integral values, of their anomeric ^1^H's in the 1D ^1^H NMR spectrum (Fig. 1*C*), show a fractional distribution of 0.3:0.23:0.23:0.23 for A–D α-Fuc units. Through NOE assignments, α-Fuc unit B shows an inter-residue connection B1–D4 with δ_H_-δ_H_ at 5.47 to 4.15 ppm (gray spectrum, Fig. 2*A*), which is indicative of a glycosidic bond between the α-Fuc unit B and unit D at its C4 position. The low-field ^13^C shift (∼6 ppm) at this C4 ring site of the D unit (HSQC spectrum at Fig. 2*C*, Table 1) is consistent with glycosylation. Note that all α-Fuc units, except the B unit as expected, show an inter-residue NOE cross-peak between their anomeric ^1^H's with ^1^H3 of the GlcA unit with δ_H_ at 3.68 ppm (gray spectrum, Fig. 2*A*, Table 1). These connections clearly indicate the branching sites of the GlcA units through fucosylation.

**Figure 3:**
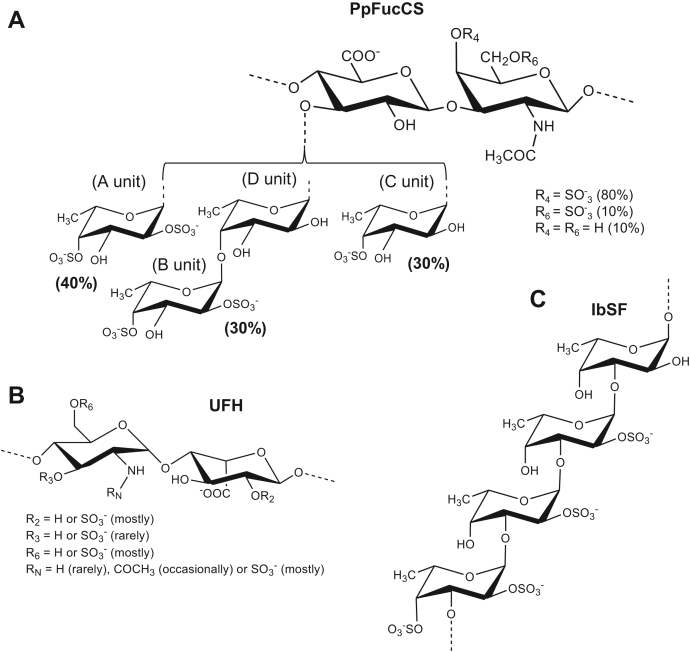
**Structural representation of sulfated glycans.***A*, PpFucCS is composed of a chondroitin sulfate backbone of alternating N-acetylgalactosamine (GalNAc) and glucuronic acid (GlcA) in repeating disaccharide units of [→3)-β-GalNAc-(1→4)-β-GlcA-(1→], where the GalNAc units are mostly 4-sulfated (80%), and, to a much lesser extent, 4,6-disulfated (10%) or nonsulfated (10%). The GlcA units are substituted at the C3 position by three types of α-fucose (Fuc) branches: 40% Fuc2,4S-(1→ (A unit in NMR), 30% Fuc2,4S-(1→4)-Fuc-(1→ (B-D units in NMR), and 30% Fuc4S-(1→ (C unit in NMR), where S = SO_3_^−^. *B*, unfractionated heparin (UFH) is mostly composed of alternating α-glucosamine (GlcN) and α-iduronic acid (IdoA) in repeating disaccharide units of [→4)-GlcN-(1→4)-IdoA-(1→]. Sulfation occurs mostly at the C6 and N-positions of GlcN and C2 position of IdoA. Although more rare, other substitutions like sulfation at the C3 position and acetylation at the N-position of the GlcN unit as well as nonsulfation in both monosaccharides can also occur. *C*, the sulfated α-fucan isolated from *Isostichopus badionotus* (IbSF) is composed of the following tetrasaccharide unit [→3)-Fuc2,4S-(1→3)-Fuc2S-(1→3)-Fuc2S-(1→3)-Fuc-(1→].

Since the α-Fuc2,4S unit denoted as B is linked to the nonsulfated unit denoted as D, a correction on the percentage of the branching units on GlcA indicates 40% Fuc2,4S (A), 30% Fuc2,4S-Fuc (B-D), and 30% Fuc4S (C). Through ^1^H–^1^H TOCSY analysis (Fig. 2*B*), three differently sulfated GalNAc units were identified: nonsulfated (N0S), 6-sulfated (N6S), and 4-sulfated (N4S) units of anomeric ^1^H's with *δ*_H_ at 4.71, 4.65, and 4.53 ppm, respectively (Table 1). Peak integration of their NMR anomeric ^1^H's shows a ratio of 1:1:8 for N0S:N6S:N4S, indicating 80% and 10% sulfation substitution, respectively, at C4 and C6 positions of the GalNAc units. This demonstrates that the CS backbone of PpFucCS is mostly CS-A (4-sulfated). The chemical structure obtained by NMR for PpFucCS is provided in Figure 3*A*.

### Structure, anti-SARS-CoV-2, and cytotoxic effects of sulfated glycans

**Figure 4:**
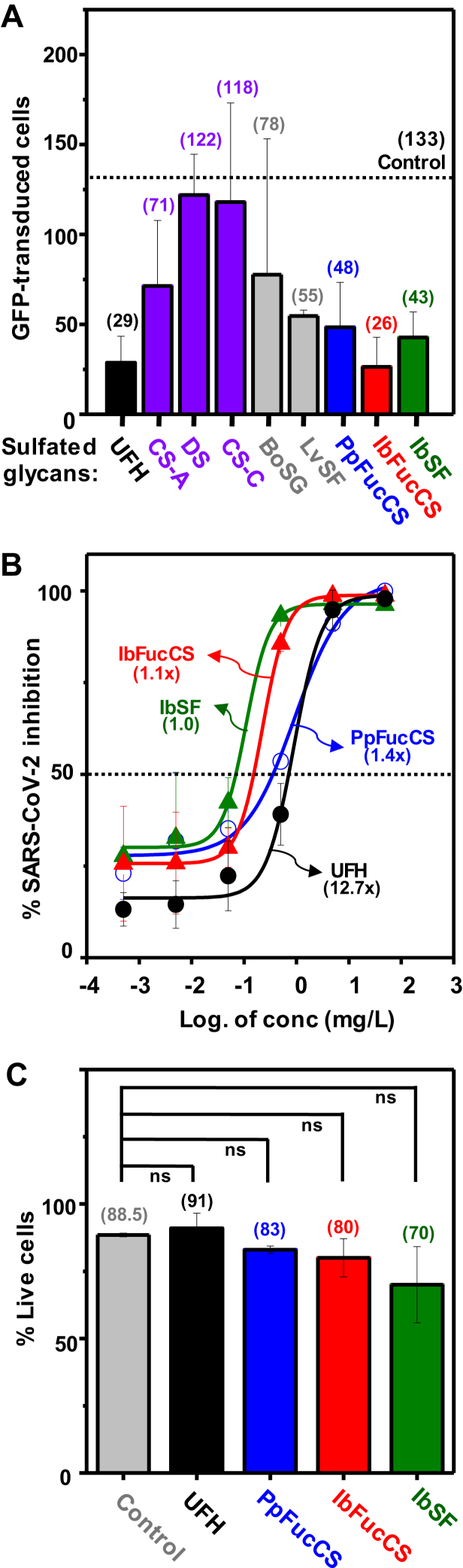
**Anti-SARS-CoV-2 activity of different sulfated glycans.** Plots showing (*A*) quantitation of GFP-transduced cells in the presence of different mammalian GAGs: unfractionated heparin, UFH (*black*); chondroitin sulfates CS-A, CS-B (dermatan sulfate), and CS-C (*purple*); and marine sulfated glycans: algal BoSG and sea urchin LvSF (*gray*), and holothurian PpFucCS (*blue*), IbFucCS (*red*), and IbSF (*green*) at an examined concentration of 50 mg/l. The average for the vehicle (control) is indicated with a *dotted line*. The numbers between parentheses on top of each bar indicate the average value. *B*, curves show percentage of SARS-CoV-2 inhibition of the three holothurian sulfated glycans and unfractionated heparin in a concentration-dependent manner. Numbers in parentheses indicate the increasing times observed in the IC_50_ values of the curves compared with the curve of the lowest IC_50_ value. *C*, plots showing percentage cell viability of HEK293T cells when treated with holothurian sulfated glycans at the highest concentration of 50 mg/l. ns stands for nonsignificant.

The ability of PpFucCS and eight mammalian and marine sulfated glycans (single concentration of 0.05 g/l) to inhibit green fluorescent protein (GFP) transduction in HEK293T cells infected with pseudotyped SARS-Cov-2 spike glycoprotein (SGP) on a baculovirus vector was investigated (Fig. 4). Among all mammalian GAGs tested, UFH (structure in Fig. 3*B*) exhibited an inhibition potency of 78% as compared with the control (black bar in Fig. 4*A*), whereas the series of chondroitin sulfates was much less active (purple bars, Fig. 4*A*).

The marine sulfated glycans studied as SARS-CoV-2 inhibitors include the sulfated galactan from the red alga *Botryocladia occidentalis* (BoSG), [3-β-Gal2R_1_4R_2_-(1→4)-α-Gal2R3R-(1→]_n_, in which R = SO_3_^−^ or OH, R_1_ and R_2_ = 66% and 33% sulfation, respectively (53); the sulfated fucan from the sea urchin *Lytechinus variegatus* (LvSF), [→3)-α-Fuc2,4S-(1→3)-α-Fuc2S-(1→3)-α-Fuc2S-(1→3)-α-Fuc4S-(1→]_n_ (54) (gray bars, Fig. 4*A*); PpFucCS (blue bar, Fig. 4*A*); *I. badionotus*-derived FucCS (IbFucCS) (red bar, Fig. 4*A*); a FucCS molecule composed of 4% α-Fuc4S (like unit C of PpFucCS, Fig. 3*A*) and 96% α-Fuc2,4S (like unit A of PpFucCS) as branching monosaccharides and backbone GalNAc units mostly 4,6-di-sulfated (51); and an *I. badionotus*-derived sulfated fucan (IbSF) (structure in Fig. 3*C*) (green bar, Fig. 4*A*) (50).

The three holothurian sulfated glycans (PpFucCS, IbFucCS, and IbSF), which showed the highest potencies of SARS-CoV-2 inhibition at the single concentration (0.05 g/l) assuming the average values displayed between parentheses (Fig. 4*A*), were investigated further to check for a concentration-dependent response, compared with UFH (Fig. 4*B*). The concentrations used were 50, 5, 0.5, 0.05, 0.005, and 0.0005 mg/l. Half-maximal inhibitory concentration (IC_50_) and confidence limit values obtained from the resultant curves are shown in Table 2. From these results (Fig. 4*B* and Table 2), we could observe that the three holothurian sulfated glycans exhibit (i) inhibitory potencies at the lowest (starting) concentration similar to UFH, (ii) similar efficiency among them, and (iii) around 12 times more potency than UFH based on the IC_50_ values. Cytotoxic effects were not observed for the three holothurian sulfated glycans in HEK293T cells at the highest concentration analyzed (Fig. 4*C*).

**Table 2:** Summary of IC_50_ calculations for SARS-CoV-2 sulfated glycan inhibitors

Sulfated glycan	IC_50_ (mg/l)^a^	95% CI	*p*^b^	*p*^c^	*p*^d^
UFH	0.2497	0.09372–6.248	-	-	-
PpFucCS	0.0279	0.008141–0.09018	0.0149	-	-
IbFucCS	0.0217	0.006013–0.06791	0.0053	0.5840	-
IbSF	0.0196	0.005204–0.05298	0.0018	0.3999	0.9432

Abbreviation: 95% CI, 95% confidence limits.

a IC_50_ values of anti-SARS-CoV-2 inhibitory activity of heparin and holothurian sulfated glycans were determined against HEK 293T cells infected with baculovirus pseudotyped with SARS-CoV-2 (Wuhan strain) wildtype S-protein with 95% confidence limits.

b *p*-Values were determined by comparing the IC_50_ values of holothurian sulfated glycans with UFH using extra sum of squares F test.

c *p*-Values were determined by comparing the IC_50_ values of holothurian sulfated glycans with PpFucCS.

d *p*-Values were determined by comparing the IC_50_ values of holothurian sulfated glycans with IbFucCS.

### Binding properties of sulfated glycans with S-protein and N501 mutant RBDs

The quality of interactions between the S-protein and the different sulfated glycans, including the three holothurian ones and UFH, was investigated through direct binding or competitive assays using SPR with heparin immobilized at the streptavidin (SA)-coated sensor chip (Figs. S4, S5, and 5). First, the interaction of both S-protein (Fig. S4*A*) and N501Y mutant (Fig. S4*B*) RBDs was examined. From these sensorgrams, kinetic values such as the association rate constant (k_a_), dissociation rate constant (k_d_), and dissociation constants (K_D_) for the studied intermolecular complexes were generated (Table S1). Results have indicated that S-protein and N501Y mutant RBDs bind to heparin with K_D_ values of 94 and 1800 nM, respectively.

**Figure 5:**
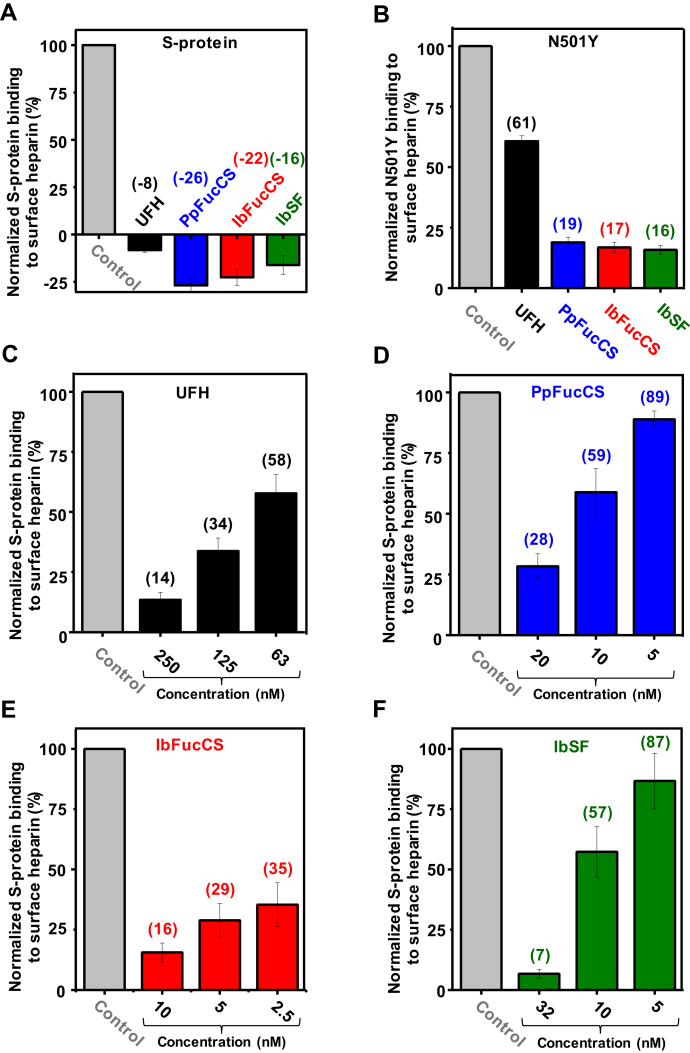
**SPR bar plots.** The bar plots (based on triplicate experiments for standard deviation) indicate normalized S-protein (*A* and *C–F*) or N501Y mutant (*B*) binding to surface heparin inhibited with different sulfated glycans and concentrations. Control bars are in *gray*, UFH in *black*, PpFucCS in *blue*, IbFucCS in *red*, and IbSF in *green*. The numbers on top of each bar (except for control bars used for normalization) indicate the average value obtained in the experiments.

Second, results from an SPR solution competitive analysis of all four sulfated glycans together with S-protein RBD (Figs. S5*A* and 5*A*) and N501Y mutant RBD (Figs. S5*B* and 5*B*) have indicated that all marine sulfated glycans, PpFucCS, IbFucCS, and IbSF, are potent in inhibiting the interactions of both RBDs onto the heparin surface. Strong inhibition was observed in the experiment with S-protein as seen from the negative values in the sensorgrams (Fig. S5*A*) and in the related bar plots (Fig. 5*A*). Inhibition of binding of N501Y mutant RBD to a heparin surface by all three holothurian sulfated glycans was stronger than by UFH (Figs. S5*B* and 5*B*). A brief explanation for the negative SPR values is shown at Figure S6.

Third, changes of SPR response units in a concentration-dependent manner were measured for each of the four sulfated glycans in the presence of the S-protein RBD (Figs. S5, *C–F* and 5, *C–F*). The IC_50_ values (12.3, 1.9 and 12.2 nM) obtained using these curves demonstrate that all three holothurian sulfated glycans interact more strongly with S-protein RBD than UFH does (76.7 nM) (Table 3). The IC_50_ values observed for sulfated glycan binding to S-protein RBD were ∼6 times lower for IbSF and PpFucCS than UFH but 25 times lower for IbFucCS than UFH. These data, derived from the SPR analyses, agree well with the greater inhibition by the three holothurian sulfated glycans as compared with UFH as seen in the concentration-dependent response curves against SARS-CoV-2 (Fig. 4*B*).

**Table 3:** IC_50_ values (nM) of holothurian sulfated glycans required to inhibit binding of heparin to SARS-CoV-2 S-protein RBD

Sulfated glycan	IC_50_ (nM)^a^
UFH	76.7 ± 12.3
PpFucCS	12.3 ± 2.5
IbFucCS	1.9 ± 0.1
IbSF	12.2 ± 3.8

a Values were determined from SPR measurements by examining the binding of S-protein RBD to surface heparin in competition with UFH and the holothurian sulfated glycans. Standard deviations (±SD) are determined from triplicated SPR measurements.

### Molecular dynamics

**Figure 6:**
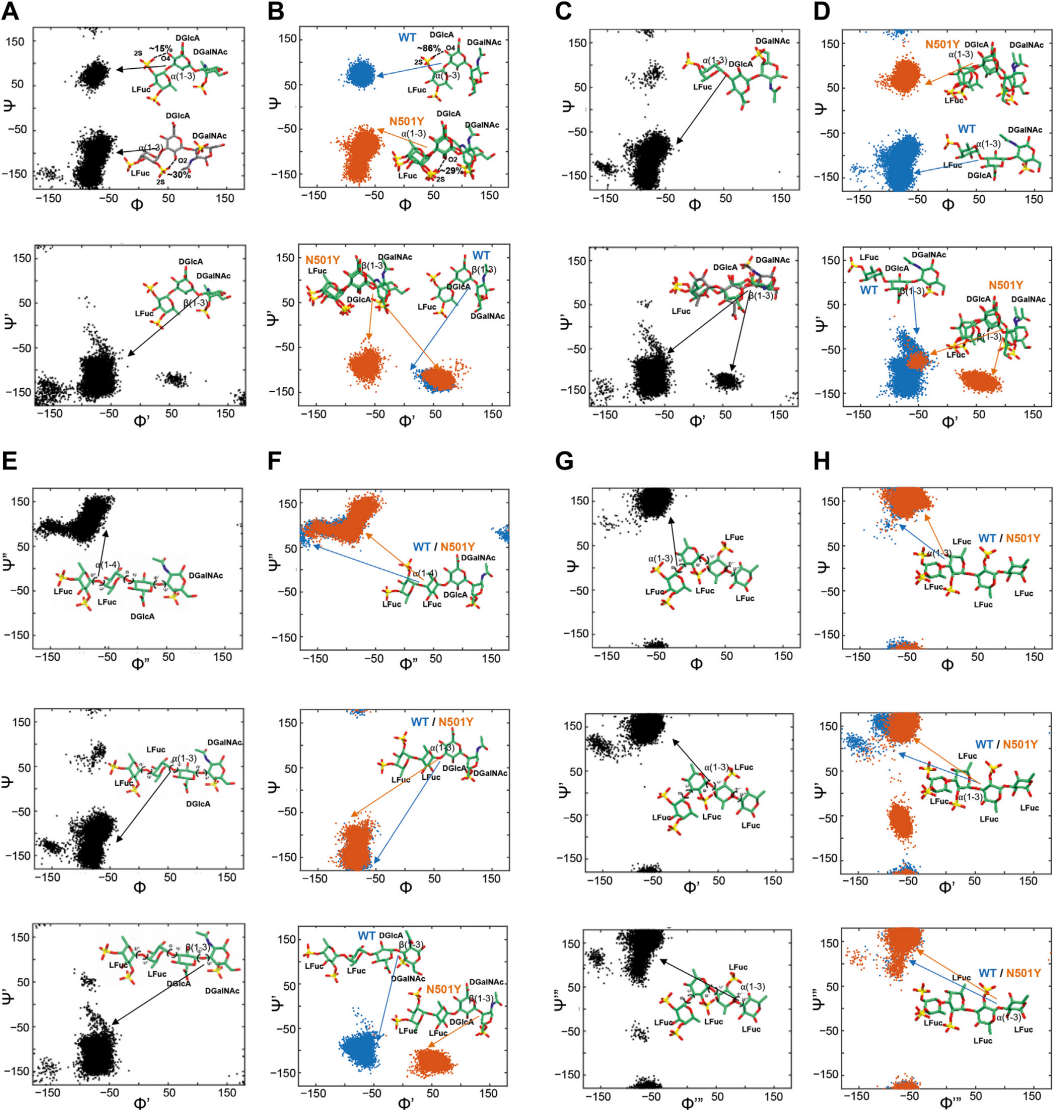
**Molecular modeling of the oligosaccharide building blocks from the holothurian sulfated glycans in their unbound and bound states with SARS-CoV-2 S-protein RBD.***A* and *B*, PpFucCS1, (*C* and *D*) PpFucCS2, (*E* and *F*) PpFucCS3, (*G* and *H*) IbSF. *A*, *C*, *E*, and *G*, 2D plots of the distribution of glycosidic torsion angles of the marine glycans in their unbound state, from 1 μs MD simulations. *B*, *D*, *F*, and *H*, 2D plots of the distribution of glycosidic torsion angles of the marine glycans in their bound state with S-protein RBD, from 200 ns MD simulations. 3D conformations of the composing oligosaccharide structures of the holothurian sulfated glycans are shown in sticks. Bound glycan glycosidic torsional plots are shown in *blue* (WT) and *orange* (N501Y).

The initial energy-minimized 3D structures of heparin and four oligosaccharide building blocks derived from the holothurian sulfated glycans (PpFucCS, IbFucCS, and IbSF; see constructs in Experimental procedures) were prepared in GLYCAM-Web (glycam.org) (55). The conformations of the free states of the four oligosaccharide building blocks, three from *P. pygmaea*, PpFucCS1 (which is also found in IbFucCS), PpFucCS2, and PpFucCS3, and the one from *I. badionotus* (IbSF), were obtained from extensive 1 μs MD simulations of each compound in water (Fig. 6). Table S2 lists the dihedral angles for the glycosidic linkages. The trisaccharide PpFucCS1 exhibited two major minima at the α(1→3) glycosidic linkage and one minimum at the β(1→3) glycosidic linkage (Fig. 6*A*). The trisaccharide PpFucCS2 showed one global minimum at the α(1→3) glycosidic linkage and two minima at the β(1→3) glycosidic linkage (Fig. 6*C*). 3D conformations corresponding to the global minima are shown within each dihedral plot in Figure 6. We also observed that the sulfation pattern of the Fuc ring in the trisaccharides had an effect on the formation of an intramolecular interaction between Fuc and the neighboring GlcA. The sulfate group at the 2-position of Fuc formed intramolecular hydrogen bond interactions with hydroxyl groups at the 2- and 4-positions of the neighboring GlcA. The pairwise interaction of the 2-sulfate of Fuc with the 4-hydroxyl and with the 2-hydroxyl of GlcA were maintained for ∼15% and ∼30% of the simulation time, respectively. In contrast, the sulfate group at the 4-position of Fuc did not exhibit such interactions. The two tetrasaccharides (PpFucCS3 and IbSF) showed distinct single minima for the α(1→4), α(1→3), and β(1→3) glycosidic linkages, corresponding to a single major conformation (shown as the 3D structures of the glycans in Fig. 6, *E* and *G*).

### Molecular docking and MD simulations of S-protein–glycan complex

**Figure 7:**
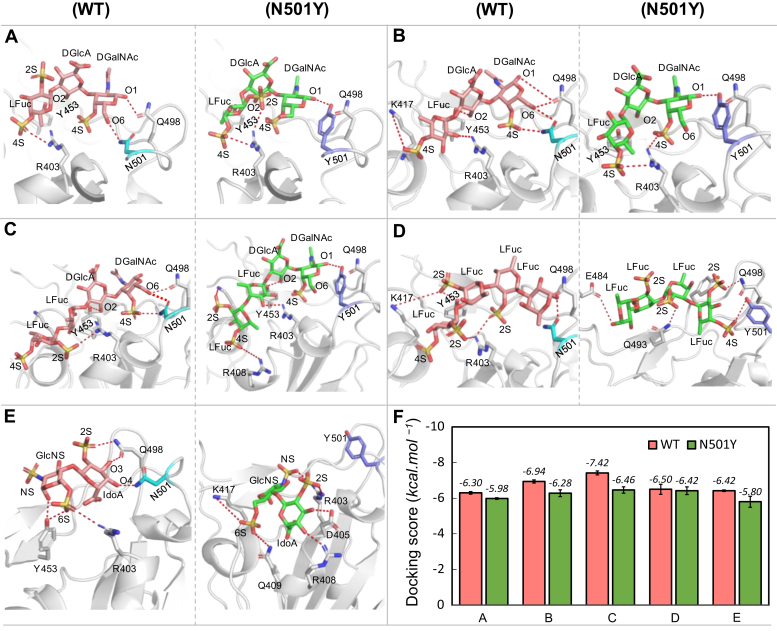
**Predicted binding poses of sulfated glycans bound to wildtype (*left panel*) and N501Y mutant (*right panel*) of SARS-CoV2 S-protein RBD.** Docked glycan in WT (*pink*) and in N501 mutant (*green*) and selected neighboring interacting residues (*gray*) are shown in sticks for (*A*) PpFucCS1, (*B*) PpFucCS2, (*C*) PpFucCS3, (*D*) IbSF, and (*E*) heparin. *Dashed lines* indicate polar interactions between the RBD and glycan. *F*, average docking scores from five independent docking runs as obtained from AutoDock Vina. Error bars represent ±standard deviation.

A potential GAG-binding site for a heparin disaccharide near N501 in the S-protein was identified by conducting a docking search centered on residue Y453 (Fig. 7), as explained in Experimental procedures. Figure S7 shows the structure of the trimeric SARS-CoV-2 S-protein, including the location of the binding site that is studied in this work, which is situated on the open conformation RBD of one monomer. The best-scoring docked pose of the heparin disaccharide (Fig. 7*E*) predicts that heparin binds to a site close to the N501Y mutation site. In the WT RBD, the O3 and O4 hydroxyl groups of IdoA participate in hydrogen bond interactions with the side chains of Q498 and N501, respectively. However, in the N501Y mutant, IdoA binding differs from WT and is oriented toward R408, where the O3 and O4 hydroxyl groups interact with D405 and R408, respectively. Heparin binding to the N501Y mutant lacks key GlcNS(6S)–arginine interactions, which leads to a reduction in binding affinity for the mutant. Overall, the N501 and R403 residues in the RBD and the 6-sulfation of GlcNS in heparin are essential for heparin binding to this site in the S-protein RBD.

The four marine oligosaccharide building blocks were also docked into the WT S-protein RBD and its N501Y mutant using the same docking protocol. Molecular docking of the PpFucCS/IbFucCS and IbSF constructs in the binding site shows that all the molecules bind to the S-protein RBD WT and N501Y mutant in a similar binding mode (Fig. 7, *A–D*). Their corresponding docking scores (Fig. 7*F* and Table S3) indicate that the marine sulfated oligosaccharides bind stronger to the WT RBD than to the N501Y mutant (although for IbSF the difference in docking scores is smaller than the standard deviations in the docking scores obtained from multiple runs with different fixed seeds). The FucCS-derived oligosaccharides from *P. pygmaea* and *I. badionotus* (PpFucCS1, PpFucCS2, and PpFucCS3) each contain the 4-sulfation at GalNAc and Fuc units with various sulfation patterns, 2,4S in PpFucCS1 and PpFucCS3, and 4S in PpFucCS2. The sulfate groups in Fuc exhibit electrostatic interactions with the positively charged (at physiological pH) residue, R403. The GlcA is always oriented in a way that the O2 hydroxyl interacts with Y453, while COO^–^ is exposed to solvent. GalNAc, which has a 4-sulfation, is seen to interact with Q498, both in the WT and N501Y mutant. The O6 hydroxyl of GalNAc interacts with N501 in the WT, whereas Y501 does not show any polar interactions with the O6 hydroxyl. This loss of hydrogen bond interaction with the RBD is a plausible cause for the weaker binding observed experimentally in the N501Y mutant (Fig. 7*F*). Of interest, in PpFucCS1, the O6 hydroxyl of GalNAc can form a hydrogen bond interaction with the 2-sulfate group of Fuc, in the mutant-bound state, which was not observed from the relative orientation when bound to the WT (Fig. 7*A*).

In the docking, IbSF binds differently to the WT and mutant (Fig. 7*D*). In the WT, it binds in a manner similar to the PpFucCS oligosaccharides, where the reducing end binds to the Q498/N501 pair; but in the mutant, this subpocket is occupied by the nonreducing end. In the WT, the 2-sulfation interacts with K417/R403, but this is not observed in the mutant. Instead, polar interactions with E484, Q493, and Q498 are observed in the IbSF–N501Y complex.

The top-scoring docking poses of the marine oligosaccharide constructs in the WT and N501Y mutant were then subjected to 200 ns all-atom MD simulations with explicit solvent, in order to obtain an optimal dynamic picture of the binding mode found from docking of ligand to protein. Root-mean-squared deviation (RMSD) plots for the simulations are shown in Figure S8 and demonstrate that the conformations were maintained reasonably well throughout the latter portions of the simulations. It is important to note that the binding of highly charged sulfated GAGs to protein is not the same as for typical protein–glycan complexes. The GAGs do not often show a very stable and well-defined binding mode in molecular dynamics simulations. In general, their nonsulfated regions are flexible but sulfated regions maintain more constant electrostatic interactions with charged protein side chains.

Next, the binding conformations of the glycans were analyzed and compared with their unbound state conformation in solution. The sugar 3D conformations and distribution plots of their glycosidic linkages in the protein-bound states are depicted in Figure 6, *B*, *D*, *F*, and *H*. Table S2 lists the dihedral angles for the glycosidic bonds. The dihedral angles of the docked trisaccharides seen in the PpFucCS series show different distributions in the WT compared with the N501Y mutant. The distribution of some of the bound state dihedrals for the two PpFucCS trisaccharides [β(1→3) for PpFucCS1–mutant, α(1→3) for PpFucCS2–mutant and β(1→3) for PpFucCS2–mutant] show large differences from the global minimum conformation obtained in the unbound state (Fig. 6*A versus* Fig. 6*B*, Fig. 6*C versus* Fig. 6*D*).

Similar to what was seen for PpFucCS1 in the free state, the sulfate group of the Fuc ring in the trisaccharide formed an intramolecular interaction with its neighboring GlcA unit, in the protein-bound state (Fig. 6*B*). The 2S sulfate of Fuc formed intramolecular hydrogen bond interactions with the O4 hydroxyl of the neighboring GlcA, when bound in the WT RBD. This pairwise interaction was maintained for ∼86% of the simulation time. Similarly, the intramolecular hydrogen bond between the 2S sulfate of Fuc and the O2 hydroxyl of the neighboring GlcA was maintained ∼29% of the simulation time in N501Y. In contrast, the 4S sulfate group of Fuc did not exhibit such interaction. The tetrasaccharides, PpFucCS3 and IbSF, show similar conformations both in the free and protein-bound states (Fig. 6, *E*–*H*).

### Anticoagulation

**Figure 8:**
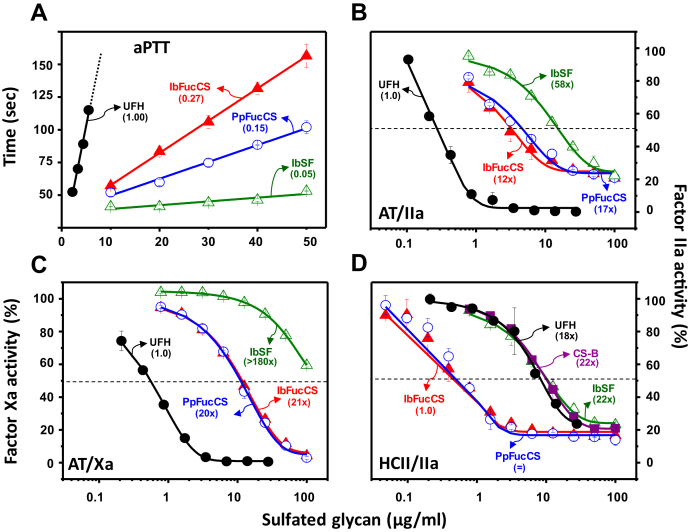
**Anticoagulant concentration-dependent response of holothurian sulfated glycans.***A*, aPTT, (*B*) AT-mediated factor IIa inhibition, (*C*) AT-mediated factor Xa inhibition, and (*D*) HCII-mediated factor IIa inhibition. The sulfated glycans tested (in a concentration range up to 100 μg/ml) were unfractionated heparin (UFH; in *black*), chondroitin sulfate B-type (CS-B, also known as DS; in *purple*), and the three holothurian sulfated glycans, PpFucCS (*blue*), IbFucCS (*red*), and IbSF (*green*). Values in parentheses in (*A*) indicate the percentage of IU/mg calculated from the aPTT curves related to the standard for heparin of 180 IU/mg. Numbers in parentheses in *B–D* indicate the increasing times observed in the IC_50_ values of the curves compared with the curve of the lowest IC_50_ value. The concentrations of coagulation (co)factors used in the experiments were 10 nM of AT or HCII and 2 nM of factors IIa or Xa.

There was a need to identify a potential anti-SARS-CoV-2 sulfated glycan devoid of anticoagulant properties since this property is commonly seen in many sulfated glycans (43). The three holothurian carbohydrates were subjected to *in vitro* anticoagulant assays, both through the activated partial thromboplastin time (aPTT) method (Fig. 8*A*) and through the catalytic inhibitions of purified serpins antithrombin (AT) and heparin cofactors II (HCII) over blood factors IIa (thrombin) and Xa (Fig. 8, *B–D*) (Table 4). The curves from the aPTT assay clearly demonstrate that all three marine sulfated glycans have weaker anticoagulant actions when compared with the international standard UFH (180 IU/mg) (black curve in Fig. 8*A*). The IU/mg values calculated from the curves of IbFucCS (red curve), PpFucCS (blue curve), and IbSF (green curve) showed reduction of effect of about 73%, 85%, and 95%, respectively, when compared with the value for UFH (Fig. 8*A*, Table 4).

**Table 4:** *In vitro* anticoagulant properties of UFH and holothurian sulfated glycans through activated plasma thromboplastin time (aPTT) and inhibition of coagulation factors IIa and Xa by antithrombin (AT) and heparin cofactor II (HCII) in the presence of sulfated glycans

Sulfated glycan	aPTT (IU/mg) a	AT/IIa	AT/Xa	HCII/IIa
		IC_50_ (μg/ml)		
UFH	180	0.28	0.55	8.7
PpFucCS	29	4.71	11.2	0.50
IbFucCS	50	3.44	11.4	0.48
IbSF	10	16.1	>100	10.6
DS	ND	ND	ND	10.6

ND, not determined.

a Values were calculated using a parallel UFH (180 IU/mg) standard curve.

As expected from the aPTT results, all three holothurian sulfated glycans exhibited lower IC_50_ values compared with UFH in the systems AT/IIa (Fig. 8*B*) and AT/Xa (Fig. 8*C*) (Table 4). The activities of both FucCS molecules (red and blue curves) were ∼15 to 20 times less active than UFH in both systems, whereas that of IbSF (green curves) was even weaker, around 60 (Fig. 8*B*) and >180 times (Fig. 8*C*) for AT/IIa and AT/Xa, respectively, less active than UFH (Table 4). Conversely, both FucCS compounds were highly active toward the less impactful HCII/IIa system of the blood coagulation (red and blue curves in Fig. 8*D*). The IC_50_ values obtained from their curves indicated around 20 times more activity than UFH (black curve), CS-B (DS) (purple curve), and IbSF (green curve) (Fig. 8*D*). In all, IbSF showed negligible anticoagulant action (green curves) as observed in all assays. As expected, based on previous findings (56, 57, 58), both FucCSs studied here showed their serpin-mediated anticoagulant action to be more pronounced toward HCII than AT.

## Discussion

### Polysaccharides from *P. pygmaea*

It is curious that among all the polysaccharides analyzed from the body wall of the sea cucumber *P. pygmaea*, a nonsulfated glycan of simple structure was found with PpFucCS. This observation is intriguing since most holothurians typically express only two sulfated glycans in their body wall, one SF and one FucCS. An example is the IbFucCS and IbSF extracted from the sea cucumber *I. badionotus* (50, 51). In addition, the structural features observed for the nonsulfated glycan is also very interesting. This polysaccharide, although slightly acidic as it binds weakly to a DEAE cationic column (elution at 0.4 M NaCl), is not composed of the common anionic monosaccharides, sialic acid and uronic acid. The multiple 1D ^1^H NMR peaks at the methyl region also suggest high content of methylation (or acetylation). Since this polysaccharide does not contain sulfation and its structure is rather simple as determined from its 1D ^1^H NMR spectrum, further attention was paid solely to the sulfated glycan PpFucCS, which was considered more likely to be of biological importance.

FucCSs are unique marine GAGs composed of repeating units of [→3)-β-GalNAc-(1→4)-β-GlcA-(1→]_n_ with α-Fuc branches attached to O3 of GlcA. These trisaccharide repeating units show variation in the composition of sulfation pattern in a species-specific manner (48). Generally, the FucCS structures reported to date have been classified into FCS type I composed of monofucosyl branches and/or FCS type II with difucosyl branches (49, 59). FucCS type I is the dominant structural form identified, whereas FucCS type II is found to be present in only a few cases so far. Examples of this less common branching unit can be found in the FucCS molecules from *Holothuria grisea* such as α-Fuc-(1→2)-α-Fuc3S (60), *Eupentacta fraudatrix* such as α-Fuc-(1→2)-α-Fuc3,4S (61), and *Holothuria lentiginosa* such as α-Fuc-(1→3)-α-Fuc4S (49). A recently identified FucCS from *Stichopus japonicus* also bears a difucosyl branch α-Fuc4S-(1→3)-α-Fuc2,4S, which is similar to PpFucCS in terms of presence of 2,4S sulfation pattern; however, it differs in interfucosyl linkage and bears a 4S sulfation in the unit (62).

The structure of PpFucCS, as fully characterized by NMR spectroscopy, is heterogenous and composed specifically of two types of monofucosyl α-Fuc2,4S and α-Fuc4S branches and one difucosyl branch α-Fuc2,4S-(1→4)-α-Fuc attached to a predominantly CS-A-type backbone. The PpFucCS structure is unique and can be classified as a mixture of FucCS type I and FucCS type II. The great abundance of the branching α-Fuc2,4S residue in PpFucCS indicates potential anticoagulant activity as this unit has been suggested as the main anticoagulant motif of this class of sulfated glycans (48, 63, 64).

### Anti-SARS-CoV-2 properties of holothurian sulfated glycans

The SARS-CoV-2 virus is densely covered on its surface with a glycosylated S-protein, also called spike glycoprotein (SGP) or S-protein, which binds to the host cell surface HS proteoglycans (6, 7, 65), consequently triggering the cascade of events leading to its entry inside the cell and establishment of infection.

Studies suggest that the abrogation of S-protein and HS binding, by exogenous administration of heparin and heparin mimetics, is capable of inhibiting SARS-CoV-2 infectivity (7, 27, 44, 46). When we examined the anti-COVID-19 activity of unique marine GAGs (PpFucCS and IbFucCS) and GAG mimetics (IbSF, LvSF, and BoSG), we found that all sulfated glycans exhibit considerable anti-SARS-CoV-2 activity compared with UFH. Holothurian PpFucCS and IbFucCS were found to exhibit similar activities despite their associated structural differences. Of interest, the heavily sulfated BoSG, a disaccharide repeating homopolymer of galactose, does not seem to affect the anti-SARS-CoV-2 activity significantly more highly, owing to its larger sulfation content or different monosaccharide type, if compared with the Fuc-containing marine polymers.

There have been reports suggesting the binding of SARS-CoV-2 to HS being regulated in a sulfation-dependent manner (34, 65, 66, 67, 68). We, however, could not observe significant differences in the anti-SARS-CoV-2 activities of the marine IbSF and LvSF, which are both homopolymers of α-Fuc units being slightly different in the sulfation patterns in their tetrasaccharide repeating units. LvSF (pentasulfated/tetrasaccharide) is 4-sulfated at the unsulfated α-Fuc unit found in IbSF (tetrasulfated/tetrasaccharide) (50, 54). It is clear to note that the addition of this 4-sulfation in the tetrasaccharide repeating unit of LvSF has no significant additive effect on its anti-SARS-CoV-2 activity.

Structure–activity relationship studies of marine sulfated glycans, in general, indicate a direct correlation between MW and activity (69, 70, 71). The MW distribution of PpFucCS is lower than that of the other holothurian glycans from *I. badionotus*. An abundant structural component of PpFucCS is the fucosyl branches, and this component has great contribution in the polydisperse bands between ∼10 and ∼60 kDa observed in PAGE. This implies that the chain length of PpFucCS is significantly shorter than that of IbSF and IbFucCS. However, the anti-COVID-19 action of all three marine sulfated glycans is still nearly equal. This suggests that there is a specific structural feature in PpFucCS that should compensate the contribution of the shorter PpFucCS backbone in its anti-SARS-CoV-2 activity. The presence of difucosyl branches with the α-Fuc2,4S unit at the terminal is the major singular feature found in the PpFucCS structure. We believe this feature might be a contributing factor to the anti-COVID-19 action of PpFucCS. However, more detailed studies regarding this structure-activity relationship are still needed to confirm this hypothesis.

The monosaccharide type and sulfation content also influence the anti-SARS-CoV-2 activities of mammalian GAG. UFH, which is mostly a trisulfated/disaccharide and contains glucose-based hexosamine (GlcN), shows greater action even with lower MW distribution (15 kDa on PAGE) as compared with the series of CS molecules (MW ranging from 20 to 60 kDa, CS-B ∼20 kDa, CS-A ∼40 kDa, and CS-C ∼60 kDa), which contains either disulfation (CS-B) or monosulfation (CS-A and CS-C) per disaccharide, and all contain galactose-based hexosamine (GalNAc). Comparing the activity of CS with FucCS corroborates the fact that the presence of α-Fuc branches (mainly the α-Fuc2,4S units) in IbFucCS and PpFucCS is important in imparting higher anti-SARS-CoV-2 activity, otherwise not observed for the CS series.

### Sulfation pattern impacts on the hydrogen bond network of the Fuc units and neighboring GlcA

As reported previously (72), Fuc makes hydrogen bond interactions with the GlcA unit to which it is linked, and this interaction seems to impact the resultant biological functions of FucCS. Hence, we used molecular dynamics simulations to examine how the quality of those hydrogen bonds between Fuc and GlcA are modulated by sulfation patterns of Fuc units (Fig. 6), particularly those found in PpFucCS (Fuc2,4S, Fuc4S, and Fuc0S) and, consequently, in IbFucCS as well (96% Fuc2,4S, and 4% Fuc4S). Hydrogen bond interactions of Fuc and GlcA are stabilized when sulfation of the Fuc unit occurs at the C2 position so that connectivities can be built with the hydroxyl groups of C2 and C4 positions in GlcA. This hydrogen bond network is lost when sulfation of Fuc units happen at the C4 position. This observation clearly indicates that Fuc sulfation patterns specifically affect the inter-residue connections with the neighboring GlcA in the 3D solution structures of the FucCS-derived oligosaccharides. Heterogeneity in terms of sulfation patterns of the composing units within oligosaccharides will ultimately impact the overall hydrogen bonding network and conformation of these molecules in solution.

### Binding properties of holothurian sulfated glycans to SARS-CoV-2 SGPs

Sulfated glycans were shown to exhibit SARS-CoV-2 inhibition by binding to its spike protein. Spike proteins are glycoproteins and classified as type I fusion proteins present on the surface of the virus and assembled as trimers (73). The S-protein has a GAG-binding domain that shows preference to bind to GAG and GAG-like molecules (28, 34). These GAGs are reported to competitively prevent the spike protein attachment to HS proteoglycans, thus preventing primary interaction with the host cell surface. Studies indicate that the heparin-binding site on the S-protein RBD is proximal to the RBD–ACE2-binding site.

Kim *et al*. (28) characterized the binding of heparin within the S-glycoprotein using a 3D model of the S-glycoprotein trimer, in which one of the three RBDs is in the “open” conformation. Such a conformation allows higher accessibility of basic residues for ligand binding. Kim *et al*. observed that HS undergoes interactions with residues at “binding site 1.” The binding site we evaluated in our current investigation through computational tools is similar to Kim *et al*.'s binding site 1, but it also includes N501. The binding sites identified by Clausen *et al*. (6) are distal and also do not include N501. At the site studied here, the marine glycan building blocks compete against heparin in their interactions with the WT S-protein RBD and its N501Y mutant, showing greater affinity for the S-protein than heparin. The better competitive binding of marine glycans over heparin could be a result of intermolecular interactions observed with the sulfate groups of the Fuc ring at the nonreducing end, suggesting the importance of the sulfation pattern on the Fuc moiety.

Positively charged residues such as lysine and arginine play a crucial role in the binding of heparin/HS to proteins and are seen rather frequently in the heparin-binding domains of proteins (74). The existence of electrostatic interactions observed between the heparin-like marine glycans and R403 provides validation of the importance of the heparin-competitive binding site we studied. Computational studies performed in this work are in excellent agreement with the noncompetitive as well as competitive SPR binding assay findings. The all-atom MD simulations provide an atomistic view of the role of the Fuc sulfation pattern in the conformational characterization of the marine glycans in their free and bound state. The molecular docking also predicted the interactions of the building blocks of the isolated marine glycans with the SARS-CoV-2 S-protein.

All three holothurian sulfated glycans studied here exhibited strong binding to S-protein RBD and mutant spike protein N501Y RBD when examined by SPR. Comparing the IC_50_ values of the marine sulfated glycans with the one from UFH, we can see an increased binding of the holothurian sulfated glycans with the S-protein. These sulfated glycans also show better binding affinity for binding to mutant protein N501Y. Since the N501Y mutation is critical and has been shown to impart increased infectivity (24, 25, 26) to the virus by tightening its binding with the receptor, these results are highly promising. Moreover, these results are in good agreement with the IC_50_ values obtained for anti-SARS-CoV-2 activities of these glycans in the pseudotyped baculoviral system. IbFucCS, however, exhibited the highest binding with the S-protein, which could be explained based on its homogenous composition of α-Fuc2,4S units, accounting therefore for a preferential interaction with the S-protein.

### Contribution from the low anticoagulant properties of holothurian sulfated glycans having anti-SARS-CoV-2 effects

Holothurian SF (38) and FucCS (48, 75) are well known to exhibit anticoagulant actions. However, the anti-SARS-CoV-2 action of the holothurian sulfated glycans could be more beneficial because of their selectivity for this antiviral effect compared with anticoagulation. Heparin and derivatives have been shown to exhibit very good anti-COVID-19 activity (6, 27, 28, 29). However, side effects associated with heparin, such as hemorrhagic tendency and heparin-induced thrombocytopenia, are a big hurdle likely preventing their application as anti-COVID-19 therapeutics (76). This points out the value in screening of other sulfated glycans (38, 77) to find ones with lower risks of harmful effects at the concentrations at which they show beneficial anti-COVID-19 activities.

The holothurian glycans studied here exhibit promising anti-SARS-CoV-2 activity. However, the application prospects of these glycans could also depend on their potential residual anticoagulant action. FucCSs, for example, IbFucCS, are very well-known anticoagulants and execute their activity by targeting serpins, primarily HCII, and AT (56), and also intrinsic tenase complex (78). We found that PpFucCS, when examined for anticoagulant activity by aPTT and serpin-mediated (AT/HCII) inhibition of factors IIa and Xa, showed moderate anticoagulant response. The observed activity of PpFucCS, although less than that of UFH and IbFucCS, was significantly greater than that of IbSF. Because the anticoagulant mode of action requires interaction with targeting factors of the coagulation pathways, certain sulfation patterns on the marine sulfated polysaccharides have been found to be significant contributors to their activity (64). An example is the presence of the α-Fuc2,4S motif in anticoagulation (63, 64, 79). The PpFucCS structure, although it has abundant α-Fuc2,4S units, still shows a lower percent content of this motif, –55% of all its α-Fuc units, as compared with 96% found in IbFucCS (51). This can explain the lower anticoagulant activity of PpFucCS, compared with IbFucCS as observed in our study and reported earlier (80).

IbSF, as reported earlier (50) and herein, shows, among all marine sulfated glycans thus far examined, the lowest anticoagulant action. On the path toward development of an optimal anti-SARS-CoV-2 sulfated glycan with significant S-protein binding and potent SARS-CoV-2 inhibitory effect, but having minimal anticoagulant side effects, IbSF provides a good lead for future research to develop an effective and selective new anti-COVID-19 agent.

### Concluding remarks

In conclusion, our work led to the identification of a new and unique FucCS with both difucosyl and monofucosyl branches in its structure. Anti-COVID-19 analyses of new marine sulfated glycans revealed promising anti-COVID-19 effects primarily mediated *via* competitive inhibition of S-protein binding with the host cell surface HS proteoglycans. Our results suggest the possibility to be able to obtain anti-SARS-CoV-2 benefits from marine sulfated glycans with lower side effects, and the best example of that we report is the sulfated fucan from *I. badionotus* (IbSF), which had strong S-protein binding but negligible anticoagulant activity.

## Experimental procedures

### Materials

Sea cucumbers *P. pygmaea* and *I. badionotus* were obtained from the Gulf Specimen Lab (Gulf of Mexico, Florida Keys). Papain, Sephadex G15 medium, and DEAE cellulose resin were purchased from Sigma. Pacific Hemostasis KONTACT Reagent, Pacific Hemostasis calcium chloride solution, and Pacific Hemostasis universal coagulation reference plasma were purchased from Thermo Fisher Scientific. Coagulation factors Xa, IIa, AT, and HCII were from Haematologic Technologies. Chromogenic substrates S2238 and CS-11(32) were purchased from Chromogenix (AB) and Aniara Diagnostica, respectively. UFH (180 IU/mg), CS-A, CS-B, and CS-C were from Sigma. Baculovirus pseudotyped with SARS-CoV-2 (Wuhan strain) WT S-protein containing a GFP reporter was obtained from Montana Molecular. HEK293T cells were obtained from ATCC. SARS-CoV-2 S-Protein RBD mutant (N501Y) was purchased from Sino Biological. Amine-PEG3-Biotin was from Pierce. Sensor SA chips were from GE healthcare. SPR measurements were performed on a BIAcore 3000 operated using BIAcore 3000 control and BIAevaluation software (version 4.0.1). Deuterium oxide (D_2_O) (D 99.90%) was purchased from Cambridge Isotope Laboratories, Inc. NMR tubes (3 mm) were purchased from VWR International. BoSG and LvSF were previously isolated in our laboratory (53, 54).

### Extraction of holothurian sulfated glycans

The sulfated glycans IbSF, IbFucCS, and PpFucCS were isolated from the body walls of the sea cucumbers *I. badionotus* and *P. pygmaea* following a slightly modified protocol to that reported (50, 51). Briefly, the dry body wall of the sea cucumber was digested using papain (0.1 mg/100 mg of dry tissue), 5 mM cysteine, and 5 mM EDTA in 0.1 M sodium acetate buffer, pH 6.0 (2 ml/100 mg of dried tissue) at 60 °C for 24 h. The digested mixture was centrifuged (4000 rpm for 30 min), and the supernatant was precipitated using two volumes of 95% ethanol at –20 °C. After 24 h, a precipitate was obtained by centrifugation at 4000 rpm for 30 min. The precipitate was dissolved in water and dialyzed three times against distilled water prior to lyophilization to obtain the dry extract.

### Purification of holothurian sulfated glycans

The dry crude extract (20 mg) was subjected to an anion exchange chromatography (DEAE-cellulose packed) column (1 × 20 cm). Polysaccharides were eluted and separated using a linear gradient of NaCl (in 0.1 M NaOAc, pH 6.0) from 0 to 3 M at a flow rate of 18 ml/h. The obtained fractions of all the polysaccharides were monitored by 1,9–dimethylmethylene blue assay (81). Purified fractions of PpFucCS were also assayed for the presence of hexoses (82), uronic acids (83), and sialic acids (84) by the Dubois reaction, Carbazole reaction, and Ehrlich assay, respectively. The NaCl concentration was estimated by conductivity. The polysaccharide fractions were pooled and dialyzed three times against water and lyophilized. The dialyzed sugars were further purified on size exclusion column Sephadex G15 (1 × 30 cm).

### MW determination

The MW of the purified PpFucCS was determined by running native PAGE along with MW standards: LMWH (∼8 kDa), UFH (∼15 kDa), CS-A (∼40 kDa), CS-C (∼60 kDa), and native sulfated glycans IbFucCS and IbSF (85). Sample amount of 10 μg (in 50% glycerol, 0.5 M Tris, pH 6.8) was loaded on a 1-mm-thick discontinuous PAGE system having 4% stacking gel and a 12% resolving gel phase. Electrophoretic migration was achieved at 100 V in 0.25 M Tris-Glycine running buffer, pH 8.3. The migration of bands was tracked by 0.02% bromocresol green dye added to one of the lanes of the gel. The gel was stained using 0.1% (w/v) toluidine blue (in 1% acetic acid) for 1 h. Destaining of the gel was done using 1% acetic acid.

### Nuclear magnetic resonance

The NMR sample of PpFucCS and nonsulfated glycan was prepared by dissolving 9.8 mg of pure polysaccharide in 200 μl D_2_O (99.90%). A series of 1D ^1^H and 2D ^1^H–^1^H homonuclear or ^1^H–^13^C heteronuclear NMR spectra were recorded, which included COSY, TOCSY, NOESY, and HSQC spectra. All NMR spectra of PpFucCS were acquired at 50 °C on 600 MHz Bruker Avance III HD using a 5-mm BBFO RT probe equipped with Z gradient. ^1^H-^1^H COSY and ^1^H-^1^H TOCSY spectra were acquired using T_1_ and T_2_ acquisition times of 0.06 and 0.243 s, respectively, and a total of 512 number of scans were used for the complete acquisition. Obtained free induction decay's for TOCSY were processed by zero filling and linear prediction prior to Fourier transform. A series of ^1^H–^1^H NOESY spectra were acquired with different mixing times (25, 50, 100, 150, and 200 ms) using T_1_ and T_2_ acquisition times of 0.03 and 0.243 s, respectively. The NOESY spectrum at 150 ms mixing time was used for the assignments of the NOE cross-peaks of PpFucCS. The ^1^H-^13^C HSQC spectrum of PpFucCS was acquired using T_1_ and T_2_ acquisition times of 0.121 and 0.005 s, respectively, using 1024 × 256 points. HSQC acquisition was performed *via* double insensitive nuclei enhancement by polarization transfer using Echo/Antiecho TPPI gradient selection with decoupling during acquisition and using trim pulses in insensitive nuclei enhancement by polarization transfer. Data were processed by zero filling and linear prediction prior to Fourier transform. In all 2D NMR experiments, delays of five times T_1_ relaxation times were included between multiple pulses to ensure full recovery of magnetization during the experimentation. Acquired NMR data were further processed and analyzed using MestreNova 14.1.0 and TopSpin 4.0 software.

### SARS-CoV-2 pseudotype virus

HEK 293T cells expressing Ace2 (86) were plated in 12-well tissue culture dishes and infected with the baculovirus pseudotyped with SARS-CoV-2 (Wuhan strain) WT S-protein containing GFP reporter (#C1110G, Montana Molecular) with serial dilutions of the stock (10^2^ to 10^7^) (87, 88, 89). The calculation of virus titers was done by enumerating GFP-positive transduced cells in a dilution under a fluorescence microscope (EVOS-FL, Thermo Fisher Scientific) and multiplying by the dilution factor and the volume plated.

### Virus inhibitor screening

The HEK293T cells expressing Ace2 were plated on a 96-well plate in Dulbecco's modified Eagle medium (DMEM) supplemented with 10% fetal bovine serum. The cells were incubated at 37 °C with 5% CO_2_, protected from light for 12 to 24 h. Serial dilutions of the inhibitory compounds (50, 5, 0.5, 0.05, 0.005, and 0.0005 mg/l) were made in triplicates in DMEM with the end volume of 100 μl each. The pseudotype virus stock 2.5 μl of the 2 × 10^10^ units/ml was mixed with the diluted inhibitory compounds and incubated for 1 h with 5% CO_2_ at 37 °C, which was then laid over HEK293T cells plated in the 96-well tissue culture dishes, in addition to 0.6 μl of 500 nM sodium butyrate to a final concentration of 2 mM. Plates were incubated for 60 h at 37 °C with 5% CO_2_. The cells were then fixed in formaldehyde (3.7%), and the assay was read on a Cytation 5 automated fluorescence microscope (BioTek Instruments, Inc). A nonlinear regression (curve fit) using normalized data sets (PpFucCS-UFH, IbSF-UFH, IbFucCS-UFH, PpFucCS-IbSF, PpFucCS-IbFucCS and IbSF-IbFucCS) was used to compute IC_50_ values in GraphPad Prism 9. IC_50_ values between groups were compared using extra sum-of-squares F test with *p* value <0.05 being considered significant.

### Cell viability assay

The HEK293T cells were seeded in 12-well tissue culture plates in complete medium (DMEM + 10% fetal bovine serum) and incubated at 37 °C and 5% CO_2_ till they reached confluency. The test compounds (sugars) were laid over the HEK293T cells to achieve a final concentration of 50 mg/l along with 2 mM sodium butyrate in an end volume of 500 μl for each well. After incubation for 60 h, the cells were harvested by trypsinization. The cell viability assay was performed in duplicates using the trypan blue exclusion assay and read on a TC20 automated cell counter (Bio-Rad) according to the manufacturer's protocol.

### Preparation of heparin chip for SPR

UFH (2 mg) and amine-PEG3-Biotin (2 mg) were dissolved in 200 μl H_2_O, and 10 mg NaCNBH_3_ was added. The reaction mixture was heated at 70 °C for another 24 h, and after that a further 10 mg of NaCNBH_3_ was added and the reaction was heated at 70 °C for another 24 h. After cooling to room temperature, the mixture was desalted with the spin column (3000 MW cut-off). Biotinylated heparin was collected, freeze-dried, and used for SA chip preparation. The biotinylated heparin was immobilized to the SA chip based on the manufacturer's protocol. In brief, a 20-μl solution of biotinylated heparin (0.1 mg/ml) in HBS-EP running buffer was injected over flow cell 2 (FC2) of the SA chip at a flow rate of 10 μl/min. The successful immobilization of heparin was confirmed by the observation of an ∼200 response unit increase in the sensor chip. The control flow cell (FC1) was prepared by 1-min injection with saturated biotin.

### Measurement of interaction between heparin and S-proteins using SPR BIAcore

BIAcore 3000 (GE healthcare) was used in the following SPR analysis. The S-protein samples were diluted in HBS-EP buffer (0.01 M Hepes, 0.15 M NaCl, 3 mM EDTA, 0.005% surfactant P20, pH 7.4). Different dilutions of protein samples were injected at a flow rate of 30 μl/min. At the end of the sample injection, the same buffer was flowed over the sensor surface to facilitate dissociation. After a 3-min dissociation time, the sensor surface was regenerated by injecting with 30 μl of 2 M NaCl to get fully regenerated surface. The response was monitored as a function of time (sensorgram) at 25 °C. The resulting sensorgrams were used for interaction kinetics and binding affinity determination: association rate constant (k_a_), dissociation rate constant (k_d_), and binding equilibrium dissociation constant: (K_D_) calculated as K_D_ = k_d_/k_a_, by global fitting using a 1:1 Langmuir binding model from Biaevaluation software 4.0.1 (GE healthcare).

### SPR solution competition study of sulfated glycans

A solution competition study between surface heparin and different marine sulfated glycans in solution to measure IC_50_ was performed using SPR (90). In brief, S-protein RBD (250 nM) samples were mixed with different concentrations of sulfated glycans in HBS-EP buffer and were injected over a heparin chip at a flow rate of 30 μl/min. After each run, the dissociation and the regeneration were performed as described above. For each set of competition experiments on SPR, a control experiment (only protein) was performed to make sure the surface was completely regenerated and that the results obtained between runs were comparable. Once the active binding sites on S-protein molecules were occupied by sulfated glycans in the solution, the binding of S-protein to the surface-immobilized heparin should be decreased, resulting in a reduced signal. The IC_50_ values, the concentration of competing analyte resulting in a 50% decrease in signal, measured in response units, can be calculated from the plots of S-protein binding signal (normalized) *versus* sulfated glycan concentration in solution.

### MD simulations

The program package Amber20 was used for molecular dynamics (MD) simulations. Force fields Glycam06 and Amberff14SB were used for glycans (91) and proteins (92), respectively, along with the TIP3P water model (93). The *tleap* program of the Amber20 package was used to solvate the simulation systems and neutralize them with Na^+^ and Cl^−^ ions. For constant temperature simulations, the Langevin thermostat was set to 298 K and periodic boundary conditions were used (94). A time step of 2 fs was used for the simulations, unless stated otherwise. Nonbonded interactions were kept at a cutoff of 8 Å. The Berendsen barostat was used for constant pressure simulations (95). Structural analysis and all visualization were performed using Visual Molecular Dynamics (VMD1.9.4) (96) and PyMOL (97). The constructs used for the building block oligosaccharides seen in the holothurian sulfated glycans were the following: [α-Fuc2,4S-(1→3)-β-GlcA-(1→3)-β-GalNAc4S] for PpFucCS1, [α-Fuc4S-(1→3)-β-GlcA-(1→3)-β-GalNAc4S] for PpFucCS2, [α-Fuc2,4S-(1→4)-α-Fuc-(1→3)-β-GlcA-(1→3)-β-GalNAc4S] for PpFucCS3, and [α-Fuc2,4S-(1→3)-α-Fuc2S-(1→3)-α-Fuc2S-(1→3)-α-Fuc] for IbSF. The reading frame of the sulfation pattern [2,4S-2S-2S-nonS] of the IbSF tetrasaccharide was unequivocally determined by depolymerization of the native molecule by mild acid hydrolysis, followed by fractionation and NMR structural characterization of a low MW fragment (see Fig. S9 and Table S4).

### Generation of 3D structures of holothurian sulfated glycan-derived composing oligosaccharides

The initial structures of the oligosaccharide building blocks of the holothurian sulfated glycans were generated using GLYCAM-Web (glycam.org) (55), and the energy-minimized structures were used for conformational sampling using MD simulations. An MD system was built for each glycan, as explained above. Each system was minimized for 5000 steps, followed by heating the system to 298 K at constant volume. The heating of the systems was performed for the first 9000 steps, starting with an initial temperature of 0 K and continuing to a final temperature of 298 K. For the next 1000 steps, the temperature was kept constant at 298 K at constant volume. Finally, a 1 μs MD simulation of each glycan was performed under NPT (constant particle number, pressure, and temperature) conditions. Conformational analysis was performed every 0.1 ns during the entire simulation time.

### Generation of 3D structure of mutant S-protein RBD

The 3D structure of the wildtype (WT) Spike-protein receptor binding domain (RBD) was obtained from Protein Data Bank (PDB: 6M0J) (20). Disulfide bonds between residue pairs C480–C488, C379–C432, C391–C525, and C336–C361 were added during system preparation. Geometry optimization of the solvent molecules was performed while holding the mutant protein fixed. The system was then heated to 298 K at constant volume for 100 ps, with a time step of 1 fs. The protein restraints were gradually released during 500 ps of equilibration with constant NPT. Finally, 500 ps of equilibration was performed with constant NPT, with protein α-carbon atoms restrained, and allowing everything else to move. A time step of 1 fs was used during the restrained-protein MD simulations. This equilibrated structure of the N501Y mutant was used for docking.

### S-protein–glycan complex structures

MD simulations were performed on the protein–glycan complexes obtained from molecular docking. The *tleap* program was used to prepare the S-protein RBD WT–glycan and N501Y mutant–glycan complexes for MD simulation as explained before. MD simulation steps as described in the previous section were followed for the protein–glycan complexes. Finally, all atoms were released and equilibrated for 2 ns with constant NPT followed by a production run of 200 ns with constant NPT. Conformational analysis was performed every 0.02 ns during the entire production run.

### Molecular docking

Molecular docking of the marine glycans to the S-protein RBD was performed using AutoDock Vina (98). AutoDockTools was used to keep polar hydrogens, and Gasteiger charges were added to the protein and glycans (99, 100). The studied glycans were allowed to be flexible during the molecular docking studies. Each glycan was docked into the 3D structure of WT S-protein RBD obtained from the PDB (PDB ID: 6M0J) (20) and the equilibrated 3D structure of N501Y S-protein RBD. The docking box was 35 × 38 × 35 Å^3^, with the coordinates of the oxygen atom in the side chain of the residue Y453 serving as the box center. The y-dimension of the docking box spanned the receptor binding motif of the S-protein RBD. An exhaustiveness of 5 and a seed value of 0 was used. A fixed seed value in AutoDock Vina makes the predictions deterministic, and hence the same results are expected in multiple runs. The energy range cutoff was set to 5 kcal·mol^–1^. For each calculation, 50 docking poses were obtained. The best scored docking pose of each glycan, ranked by the AutoDock Vina scoring function, was used as the starting coordinates for the MD simulation of the protein–glycan complex. The docking of each compound was repeated an additional four times using fixed seed values of 1, 5, 10, and 15, and then the average docking score from the five independent docking runs in WT and N501 were compared.

### Activated partial thromboplastin time

aPTT was performed by incubating 90 μl of plasma with 10 μl of varying concentrations of polysaccharides at 37 °C for 3 min. aPTT reagent, 100 μl, was then added to the above mixture and incubated for 5 min at 37 °C. Clotting time was measured immediately following the addition of 0.025 M CaCl_2_ (100 μl). The aPTT readout was measured in seconds. UFH (180 IU/mg) was used as a positive control. The measurements were performed on an Amelung Coagulometer KC4A.

### IIa and Xa inhibition by AT and HCII in the presence of sulfated glycans

Sulfated glycans were assayed for their serpin-mediated inhibitory activity against IIa and Xa using an effective concentration of 10 nM of AT or HCII, 2 nM of IIa or factor Xa, and 0 to 100 μg/ml of sulfated glycans in 100 μl of TS/PEG buffer (0.02 M Tris/HCl, 0.15 M NaCl, and 1.0 mg/ml polyethylene glycol 8000, pH 7.4) as reported earlier (85). Sulfated glycans (10 μl) at varying concentrations were dispensed in the 96-well microtiter plate, followed by the addition of 40 μl AT (25 nM) or HCII (25 nM). A volume of 50 μl of IIa (4 nM) or Xa (4 nM) was added in the end to initiate the reaction. The plate was then immediately incubated at 37 °C for 1 min, which was followed by the addition of 25 μl of chromogenic substrate S-2238 for IIa or CS–11(32) for factor Xa. The absorbance (Abs) was then measured at 405 nm for 300 s at an interval of 15 s. Wells without sulfated glycans served as control and IIa/Xa activity in the control was considered as 100%. The residual activity in treated wells was calculated relative to that observed in the case of control wells. Heparin (180 IU/mg) was used in all the assays as a positive control, whereas DS (CS-B) was also used as a positive control only in the HCII/IIa system.

## Data availability

All data are contained within the article.

## Supporting information

This article contains supporting information (50, 69, 102).

## Conflict of interest

The authors declare that they have no conflicts of interest with the contents of this article.
