# Supplementary material for: Structural and kinetic analyses of holothurian sulfated glycans suggest potential treatment for SARS-CoV-2 infection
Source: J Biol Chem. 2021 Sep 17;297(4):101207. doi: 10.1016/j.jbc.2021.101207 (PMC8445769; doi:10.1016/j.jbc.2021.101207)
Supplement: Supporting information [file mmc1.docx]

**SUPPORTING INFORMATION**

**Structural and kinetic analyses of holothurian sulfated glycans suggest potential treatment for SARS-CoV-2 infection**

Rohini Dwivedi, Priyanka Samanta, Poonam Sharma, Fuming Zhang, Sushil K. Mishra, Pavel Kucheryavy, Seon Beom Kim, AyoOluwa O. Aderibigbe, Robert J. Linhardt, Ritesh Tandon, Robert J. Doerksen, Vitor H. Pomin

**Figure S1.** One-dimensional (1D) ^1^H NMR spectrum (δ_H_ expansion 6.0–0.0 ppm) of the purified non-sulfated glycan recorded in D_2_O, at 25 °C, on a 400 MHz Bruker NMR instrument. Peaks assigned with asterisks in the 1D ^1^H NMR spectrum are likely solvent contaminants, based on to their very narrow lines.


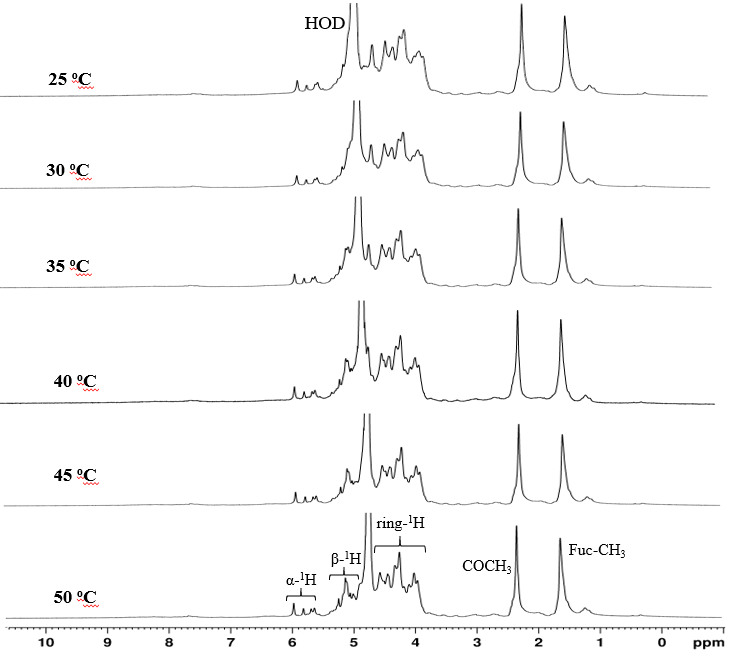


**Figure S2.** One-dimensional (1D) ^1^H NMR spectra (δ_H_ expansion from 10.6 to -1.0 ppm) of PpFucCS recorded in D_2_O, at different temperatures, on a 400 MHz Bruker NMR instrument.

**Figure S3. NOE build-up curves of PpFucCS at different mixing times.** Plotted curves show the intensities of NOE cross-peaks observed in the anomeric (^1^H1) region of fucose (Fuc) units from the NOESY spectra of PpFucCS acquired at different mixing times (25, 50, 100, 150 and 200 ms). All NOESY spectra were acquired in D_2_O at 50 °C on a 600 MHZ NMR spectrometer. Letters A-D and U denote Fuc2,4S; Fuc2,4S; Fuc4S; Fuc0S and glucuronic acid (GlcA), respectively. Numbers correspond to the respective protons within these units.

**Figure S4.** **Binding of S-protein and N501Y to surface heparin.** Sensograms showing binding of (A) S-protein and (B) N501Y mutant to surface heparin at different concentrations, as determined by surface plasmon resonance. The calculated binding affinities (dissociation constants, *K_D_*) are indicated in each panel.

**Figure S5. SPR sensorgrams from the competitive (A and B) and concentration-dependent (C-D) inhibition of different sulfated glycans on the binding of S-protein (A and C-F) and N501Y (B) to surface heparin.** (A) Concentrations of S-protein and sulfated glycans were 500 nM and 1000 nM, respectively. (B) Concentrations of the N501Y mutant and sulfated glycans were 1000 nM. (C-D) Concentration of S-protein was 500 nM. The sulfated glycans tested were unfractionated heparin (UFH), and the three holothurian polysaccharides: PpFucCS, IbFucCS and IbSF. The sulfated glycans and the tested concentrations are indicated with different colors in the panels.

**Table S1.** Kinetic values^a^ of SARS-CoV-2 S-protein RBD and N501Y mutant in interaction with heparin.

| Interaction | k_a_ (1/MS) | k_d_ (1/S) | K_D_ (M) | |
| --- | --- | --- | --- | --- |
| S-Protein RBD | 9.0×10^3^ ± 237 | 8.5×10^-4^ ± 1.7×10^-5^ | | 9.4×10^-8^ |
| N501Y RBD mutant | 2.9×10^3^ ± 237 | 5.2×10^-3^ ± 7.4×10^-5^ | | 1.8×10^-6^ |

^a^ Values were determined from SPR measurements by examining the binding of S-protein RBD and N501Y mutant RBD to surface heparin. Values with ± are the standard deviations (SD) determined from triplicate SPR measurements.

**Figure S6. SPR sensorgrams of S-protein and sulfated glycans.** (A) Original sensorgrams of S-protein RBD binding to control surface and heparin surface inhibited by different sulfated glycans. (B) Sensorgram subtracted with control signal.

Discussion: In Fig. S6*A*, the control surface (without immobilized heparin) is shown in red and the experimental surface (with immobilized heparin) is shown in blue. When S-protein was flowed over the experimental surface binding was observed by a strong increase in signal (23400-23700 RU) in the blue sensorgram on the left. With the addition of each sulfated glycan (UFH, PpFucCS, IbFucCS, and IbSF) the increase in the blue sensorgams was either greatly reduced or eliminated showing competition. The red (control) sensorgrams show little of no changes. Fig. S6*B* shows the subtraction of each control sensorgram (red) from each experimental sensorgram (blue) in green. These subtracted sensorgams are sometimes slightly negative (<50 RU) due to the noise in the measurements.**Table S2.** Average glycosidic dihedral angle (°) obtained from MD simulations of the four holothurian sulfated glycan-derived constructs. “Free” represents the glycans when solvated in water in the absence of the protein. “WT” and “N501Y” represent the glycans when they are bound to the WT and mutant RBD, respectively.

|  | PpFucCS1 | | | | PpFucCS2 | | | PpFucCS3 | | | IbSF | | |
| --- | --- | --- | --- | --- | --- | --- | --- | --- | --- | --- | --- | --- | --- |
|  | Free | WT | N501Y | | Free | WT | N501Y | Free | WT | N501Y | Free | WT | N501Y |
| Dih1  (Φ, Ψ) | −74.6,  75.2;  −76.6,  −114.8*^†^* | −72.5,  73.9 | | −69.6,  −96.9 | −78.2,  −116.3 | −79.2,  −123.3 | −70.9,  76.7 | −85.4,  110.2 | −102.9,  93.6 | −85.2,  114.8 | −69.9,  146.9 | −68.9,  138.6 | −63.0,  149.3 |
| Dih2  (Φ, Ψ) | −75.5,  −111.3 | 55.4,  −123.1 | | (59.9,  −120.0)  (−66.2,  −91.9)*^†^* | 59.4,  −123.1;  −72.7,  −107.3*^†^* | −68.8,  −97.2 | 56.8,  −121.6  −51.9,  −76.2*^†^* | −71.9,  −108.0 | −83.4,  −146.0 | −83.5,  −133.8 | −73.8,  143.7 | −84.0,  141.6 | −71.4,  152.6 |
| Dih3  (Φ, Ψ) | N/A | N/A | | N/A | N/A | N/A | N/A | −78.5,  −116.3 | −72.7,  −99.5 | 55.3,  −123.1 | −73.0,  153.6 | −65.8,137 | −69.5,  143.6 |

*^†^* Dihedral angles of a second minimum found in the MD simulation.

**Figure S7. Structure of the trimeric SARS-CoV-2 SGP (PDB ID 7DDN, used for illustration only).** The receptor binding domain (RBD) in the open conformation is colored yellow (Left). The other two RBDs are in the closed conformation. The zoomed-in image shows the top scored pose for PpFucCS3 in RBD wild-type. Protein residues enclosed within the docking grid are shown with cyan surface representation. Y453 served as the center of the docking grid.

**Table S3.** Average docking scores of the sulfated glycans from 5 independent docking runs.

| Sulfated glycan | Docking Scores^a^ | |
| --- | --- | --- |
|  | WT | N501Y |
| PpFucCS1 | −6.30 ± 0.07 | −5.98 ± 0.04 |
| PpFucCS2 | −6.94 ± 0.09 | −6.28 ± 0.19 |
| PpFucCS3 | −7.42 ± 0.11 | −6.46 ± 0.18 |
| IbSF | −6.50 ± 0.28 | −6.42 ± 0.22 |
| HS | −6.42 ± 0.04 | −5.80 ± 0.31 |

^a^ The uncertainties are shown as the standard deviations calculated from 5 independent docking runs.**Figure S8. Root mean squared deviation (RMSD) of heavy atoms of PpFucCS1, PPFucCS2, PpFucCS3 and IbSF bound to wild-type RBD and mutant N501Y.** Each plot shows the RMSD of the glycan–protein complex (prot+lig) and of the glycan (lig) only.


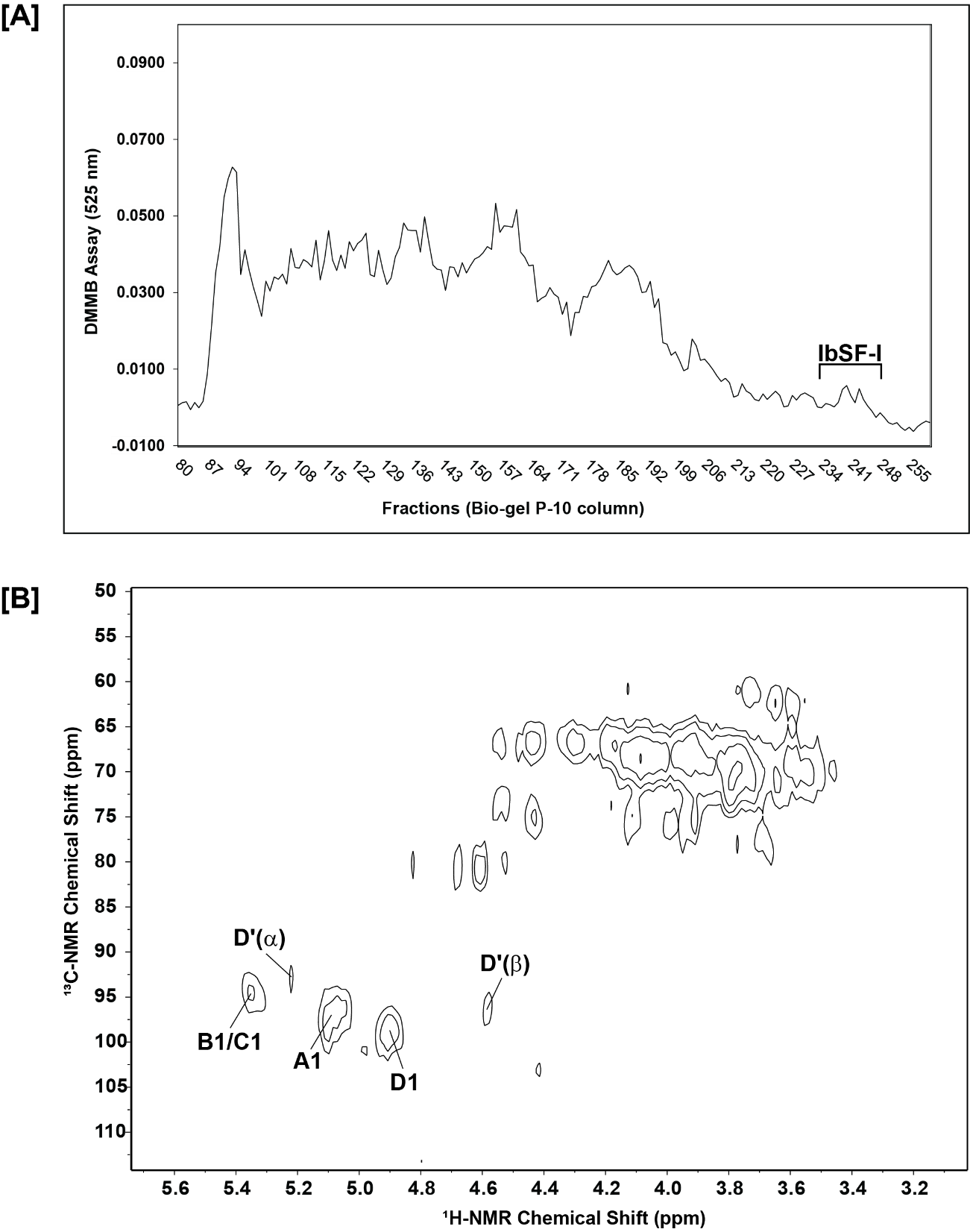


**A**

**B**

**Figure S9. Determination of the Fuc2,4S-Fuc2S-Fuc2S-Fuc oligosaccharide reading sequence of IbSF.** (A) Size-exclusion chromatography (Bio-Gel P10 column) of IbSF fragments obtained by mild acid hydrolysis at 0.05 M H_2_SO_4_, 60 ℃, 10 hours. Fractions (1 mL each) were monitored by metachromasy using 1,9-dimethylmethylene blue (DMMB) at Abs 525 nm. The fractions correspondent to the region denoted as IbSF-I were pooled, desalted and lyophilized. (B) ^1^H-^13^C HSQC spectrum of IbSF-I fraction obtained from Bio-Gel P-10 column size-exclusion chromatography. Signals assigned as A1, B1, C1, D1 and D′(α) and D′(β) are from the tetrasaccharide sequence Fuc2,4S-Fuc2S-Fuc2S-Fuc-α/β (A-B-C-D-α/β). The anomeric α/β resonances appearing at the D unit (see **Table S4**) indicate that the non-sulfated unit (D) is located at the reducing end of the IbSF-I fragment.

**Table S4.** Chemical shift (ppm) for anomeric ^1^H and ^13^C from reducing terminals of fucose units from oligosaccharides IbSF-I^a^ and references^b^. The IbSF-I was obtained by 10 hours of mild acid hydrolysis of the sulfated fucan from *Isostichopus badionotus* (IbSF).

| Units | ^1^H chemical shifts (ppm) | | ^13^C chemical shifts (ppm) | |
| --- | --- | --- | --- | --- |
|  | α-anomer | β-anomer | α-anomer | β-anomer |
| IbSF-I^a^ | **5.22** | **4.58** | **93.1** | **96.4** |
| Unsulfated fucose^b^ | **5.22** | **4.55** |  |  |
| Unsulfated fucose^b^ | **5.20** | **4.55** | **93.1** | **97.1** |
| Fucose 2-sulfate^b^ | 5.49 | 4.69 | 93.2 | 97.6 |
| Fucose 4-sulfate^b^ | 5.22 | 4.60 |  |  |
| Fucose 4-sulfate^b^ | 5.22 | 4.59 | 95.1 | 99.1 |
| Fucose 2,4-di-sulfate^b^ | 5.49 | 4.73 |  |  |

^a^ The 500 MHz ^1^H/^13^C HSQC spectrum (Figure S9*B*) was recorded at 25 ℃. Chemical shifts are referenced to internal trimethylsilylpropionic acid at 0 ppm for ^1^H and to methanol for ^13^C.

^b^ Pomin VH, Pereira MS, Valente AP, Tollefsen DM, Pavão MS, Mourão PA. Glycobiology, 2005, 15(4):369-81.

*Materials -* H_2_SO_4_ (part# A298-212, Fischer Scientific, Waltham, MA, USA) was used for the mild acid hydrolysis. Bio-Gel P-10 fine resin (part# 150-4144, Bio-Rad Laboratories, Hercules, CA, USA) and chromatography column (1.5 × 170 cm, part# 7371598, Bio-Rad Laboratories) were employed for size-exclusion chromatography. Sephadex G-15 resin (part# G15120-10G, Sigma-Aldrich, St. Louis, MO. USA) and chromatography column (1.5 × 50 cm, part# 7376607, Bio-Rad Laboratories) were used for the desalting. The fraction collector (part #2110, Bio-Rad Laboratories) was used. Peristaltic pump (part # P-1, Pharmacia Fine Chemicals, Piscataway, NJ, USA) was used to control flow rate. Samples were lyophilized at part #7522900, Labconco, Kansas, MO, USA. 1,9-Dimethyl-Methylene Blue zinc chloride double salt (DMMB) was purchased from Sigma-Aldrich, St. Louis, MO. USA (341088).

*Depolymerization and fractionation of IbSF oligosaccharides -* The mild acid hydrolysis of IbSF was employed with 0.05 M H_2_SO_4_ at 60 ℃, for 10 hours for depolymerization of *Ib*SF (30 mg) (1). Before size-exclusion chromatography, the hydrolyzed IbSF was previously analyzed by polyacrylamide gel electrophoresis (12%). Fractionation of oligosaccharides produced from IbSF was performed on a Bio-gel P-10 column (1.5 × 160 cm) eluted with 10% ethanol 1M NaCl, at a flow rate of 1 mL/15min/fraction (2). The collected fractions were measured by metachromasy using DMMB at Abs 525 nm. The population of fractions assigned as IbSF-I (Figure 3A) was pooled and desalted on a Sephadex G-15 column (1.5 × 50 cm) in distilled water, lyophilized and submitted for ^1^H-^13^C NMR analysis.

(1) Chen, S., Hu, Y., Ye, X., Li, G., Yu, G., Xue, C., and Chai, W. (2012) Sequence determination and anticoagulant and antithrombotic activities of a novel sulfated fucan isolated from the sea cucumber Isostichopus badionotus. *Biochim. Biophys. Acta*. **1820**, 989–1000

(2) Pomin, V. H., Park, Y., Huang, R., Heiss, C., Sharp, J. S., Azadi, P., and Prestegard, J. H. (2012) Exploiting enzyme specificities in digestions of chondroitin sulfates A and C: production of well-defined hexasaccharides. *Glycobiology*. **22**, 826–838

*^1^H-^13^C HSQC spectrum* **-** 2D NMR (^1^H-^13^C HSQC) spectrum was acquired on a 500 MHz Bruker AVANCE III HD with 5 mm Prodigy Cryoprobe. The IbSF-I samples (powder material after lyophilization) was dissolved in 200 µL of 99.90% D_2_O (part# 151882-1G, Sigma-Aldrich) in 3 mm NMR tube (part# SVCP-3-178, Norell, Morganton, NC, USA) with 32 scans. The acquired data were processed using the Mnova NMR software package (v. 14.2.0, MestreLab Research S.L., A Coruña, Spain).
